# Structure of WNT inhibitor adenomatosis polyposis coli down-regulated 1 (APCDD1), a cell-surface lipid-binding protein

**DOI:** 10.1073/pnas.2217096120

**Published:** 2023-05-08

**Authors:** Fu-Lien Hsieh, Tao-Hsin Chang, Sandra B. Gabelli, Jeremy Nathans

**Affiliations:** ^a^Department of Molecular Biology and Genetics, Johns Hopkins University School of Medicine, Baltimore, MD 21205; ^b^HHMI, Johns Hopkins University School of Medicine, Baltimore, MD 21205; ^c^Department of Biophysics and Biophysical Chemistry, Johns Hopkins University School of Medicine, Baltimore, MD 21205; ^d^Department of Medicine, Johns Hopkins University School of Medicine, Baltimore, MD 21205; ^e^Department of Oncology, Johns Hopkins University School of Medicine, Baltimore, MD 21205; ^f^Department of Neuroscience, Johns Hopkins University School of Medicine, Baltimore, MD 21205; ^g^Wilmer Eye Institute, Johns Hopkins University School of Medicine, Baltimore, MD 21205

**Keywords:** Wnt signaling, negative feedback regulation, extracellular domain, lipid-binding protein, X-ray structure

## Abstract

Diverse extracellular proteins negatively regulate WNT signaling. One such regulator is adenomatosis polyposis coli down-regulated 1 (APCDD1), a conserved single-span transmembrane protein. In response to WNT signaling in a variety of tissues, *APCDD1* transcripts are highly up-regulated. We have determined the three-dimensional structure of the extracellular domain of APCDD1, and this structure reveals an unusual architecture consisting of two closely apposed β-barrel domains (ABD1 and ABD2). ABD2, but not ABD1, has a large hydrophobic pocket that accommodates a bound lipid. The APCDD1 ECD can also bind to WNT7A, presumably via its covalently bound palmitoleate, a modification that is common to all WNTs and is essential for signaling. This work suggests that APCDD1 functions as a negative feedback regulator by titrating WNT ligands at the surface of responding cells.

## Significance

Adenomatosis polyposis coli down-regulated 1 (APCDD1)—a conserved single-span transmembrane protein containing a large extracellular domain—negatively regulates WNT signaling and plays important roles in hair follicle development, CNS vascular development, and glial differentiation. We report here the three-dimensional structure of the ECD of APCDD1, revealing an unusual architecture. The APCDD1 ECD consists of two closely apposed β-barrel domains (ABD1 and ABD2). ABD2 contains a large hydrophobic pocket that accommodates a bound lipid. In an in vitro assay, the ECD of APCDD1 bound to WNT7A, which contains a covalently linked palmitoleate. Collectively, the results of this study suggest that APCDD1 serves as a negative feedback regulator of WNT signaling by neutralizing WNT ligands.

WNT signaling plays a central role in embryonic development and tissue homeostasis. WNT signaling is negatively regulated by diverse extracellular and intracellular pathways (1). These include competitive inhibition of WNT–FRIZZLED binding [sFRP (2) and WIF1 (3)], blockade and/or increased turnover of LRP5/LRP6 [DKK (4), KREMEN (5), and SOST (6)], deacylation and cleavage of WNTs [NOTUM (7, 8) and TIKI1 (9), respectively], ubiquitination and degradation of FRIZZLED [ZNRF3 (10) and RNF43 (11)], and increased β-CATENIN phosphorylation and degradation [AXIN2 (12)]. Transcripts for several of these negative regulators, including AXIN2 and NOTUM, are up-regulated by WNT signaling, implying that the encoded proteins function as part of an adjustable autocrine or paracrine feedback loop.

Among the genes most consistently and most highly induced by WNT signaling is *Apcdd1* (13), which codes for a conserved single-pass transmembrane protein of ~55 kDa with a large glycosylated extracellular domain (ECD), a small cytoplasmic domain, and no discernable homology to any protein of known function. In the context of human disease, elevated WNT signaling is most prominently associated with cancer, and elevated expression of *APCDD1* has been reported in colon cancer and Ewing sarcoma cells (13–16). In transfected cells, APCDD1 inhibits WNT signaling (17–19), and, in humans, an *APCDD1* mutation (Leu9Arg in the signal peptide) is associated with hereditary hypotrichosis simplex, a defect in hair follicle development (17, 20).

In the brain and retina, WNT signaling is required for angiogenesis and vascular barrier formation, i.e., the blood–brain barrier and the blood–retina barrier. In mice, loss of *Apcdd1* causes a transient hyperplasia of the retinal vasculature, enhanced expression of *Lama2* in pericytes, and precocious development of tight junctions, an essential component of the blood–retina barrier (21, 22). Conversely, *Apcdd1* overexpression in vascular endothelial cells leads to retarded vascular growth and defective tight junctions (21). These data are consistent with a model in which APCDD1 acts as a negative regulator of WNT signaling.

APCDD1 has also been implicated in central nervous system (CNS) myelination, astrocyte migration, and adipocyte differentiation. In mice, APCDD1 promotes oligodendrocyte precursor cell (OPC) differentiation ex vivo and it enhances regenerative myelination after white matter injury (18), consistent with experiments showing that WNT signaling inhibits OPC differentiation and myelination (23). In the context of demyelinating disease, APCDD1 is increased in endothelial cells in mice with experimental autoimmune encephalitis, and APCDD1 protein and *APCDD1* mRNA are increased in human multiple sclerosis (MS) lesions (18, 24). In the embryonic chicken spinal cord, overproduction of APCDD1 promotes migration of astrocyte precursors (25). In cultured adipocytes, APCDD1 accumulates with differentiation, and siRNA-based reduction in APCDD1 inhibits adipocyte differentiation (26).

At present, the mechanism of APCDD1 action is uncertain, as APCDD1 has been variously reported to associate with LRP5, WNT3A, and β-CATENIN (17–19). To gain a mechanistic understanding of APCDD1 function, we have determined the three-dimensional structure of the ECD of APCDD1 by X-ray crystallography. The structure reveals an architecture containing two closely related β-barrel domains (ABD1 and ABD2). Structural and functional analyses show that APCDD1 has a large hydrophobic pocket in ABD2 and that APCDD1 can bind to WNT7A, presumably via its covalently bound palmitoleate, a modification that is common to all WNTs and is essential for signaling. Taken together, the results reported here suggest that APCDD1 acts as a negative feedback regulator by lowering the concentration of available WNT ligands at the surface of responsive cells.

## Results

### Protein Production and Structure Determination of the APCDD1 ECD.

**Fig. 1.**
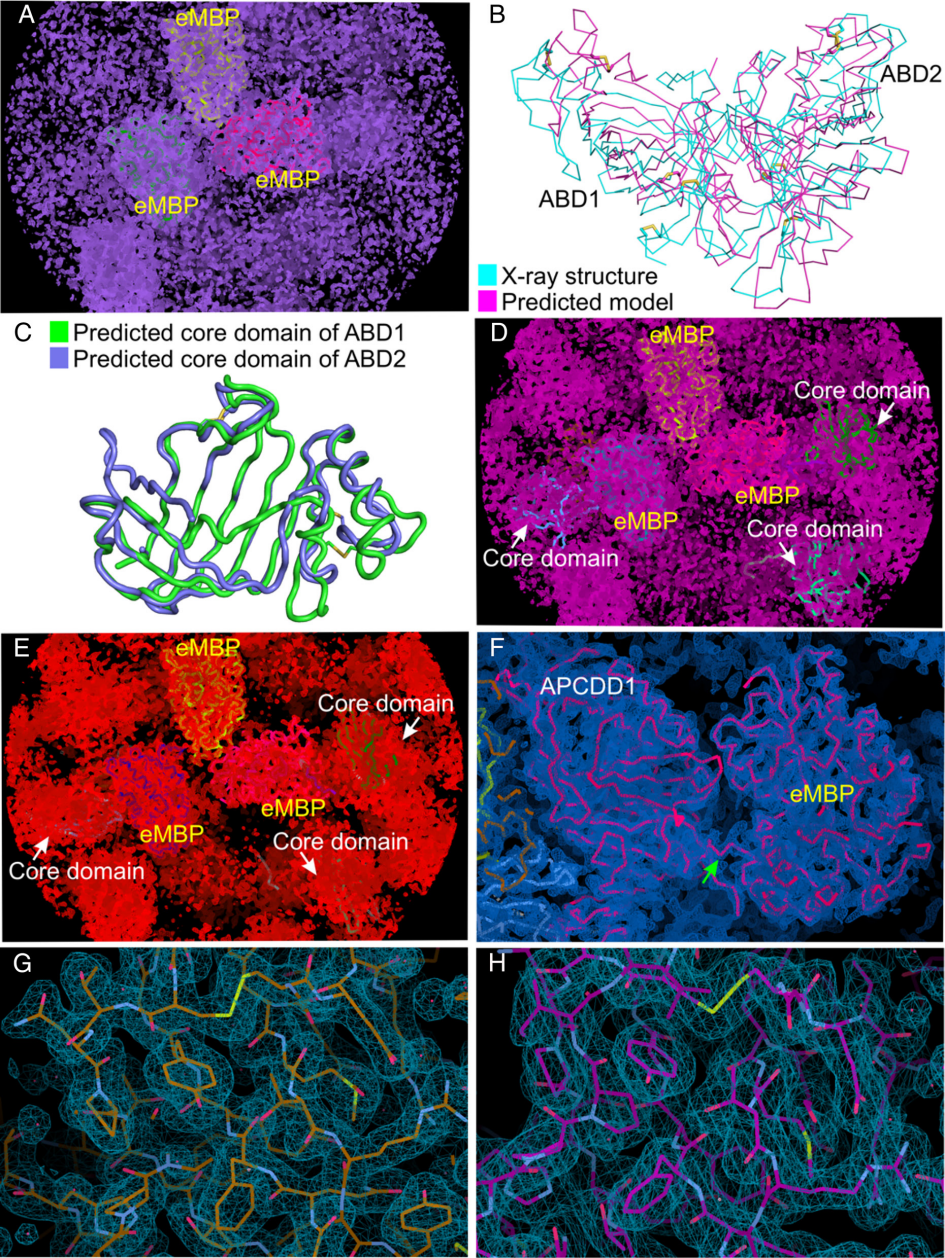
Electron density map and chain tracing of eMBP-APCDD1. (*A*) The initial electron density map (purple meshes) of eMBP-APCDD1 contoured at the 1.8 σ level after molecular replacement in PHASER using eMBP structures as the search model. Three eMBP copies (yellow, green, and magenta) fit into the electron density, whereas the electron density for APCDD1 is not interpretable. (*B*) Superposition of predicted APCDD1 model which was generated using RoseTTAFold and X-ray structure of APCDD1 (with rmsd of 3.79 Å over 358 Cα atoms) revealed high structural difference. (*C*) Superposition of predicted models of the β-barrel regions of ABD1 and ABD2, generated using RoseTTAFold, referred to as the core domain for molecular replacement. (*D*) The electron density (pink meshes) contoured at the 1.8 σ level after molecular replacement in PHASER with three eMBP copies fixed and using predicted models of core domains as search models. (*E*) The electron density modified map (red meshes) from PARROT contoured at the 1.8 σ level. (*F*) The sigmaA-weighted 2|*F*_O_|-|*F*_C_| electron density (blue meshes) after refinement in PHENIX contoured at the 1.3 σ level. The structure of the eMBP-APCDD1 fusion protein is shown as a ribbon representation (magenta). The linker between eMBP and APCDD1 is indicated by a green arrow. (*G*) The sigmaA-weighted 2|*F*_O_|-|*F*_C_| electron density (blue meshes) contoured at the 1.0 σ level. A close-up view of APCDD1 structure (chain A of crystal-form I) is shown as sticks. (*H*) The sigmaA-weighted 2|*F*_O_|-|*F*_C_| electron density (blue meshes) contoured at the 1.0 σ level. A close-up view of the APCDD1 structure (chain A of crystal-form II) is shown as sticks.

To determine the three-dimensional structure of the ECD of mouse APCDD1 (referred to hereafter simply as “APCDD1”), we produced this domain as a glycosylated secreted protein using human embryonic kidney (HEK293) cells, crystallized it, and collected X-ray diffraction data to 1.95 Å and 2.15 Å resolution from two crystal forms (*SI Appendix*, Fig. S1*A* and Table S1). Attempts to use molecular replacement for structure determination failed because of a lack of discernable homology to known structures and the low quality of predicted structures. Therefore, we produced, purified, and crystallized a fusion protein between engineered maltose-binding protein (eMBP) (27) and APCDD1 (*SI Appendix*, Fig. S1*B*), collected X-ray diffraction data to 2.3 Å resolution, and used molecular replacement with eMBP to obtain phase information (*SI Appendix*, Table S1). Although the resulting electron density map provided a good fit to the eMBP half of the fusion protein, the map of APCDD1 was of insufficient quality for model building (Fig. 1*A*).

Recognizing that the N- and C-terminal halves of APCDD1 have ~25% amino acid identity and similar predicted secondary structure patterns, and, therefore, that APCDD1 very likely consists of two domains with conserved three-dimensional structures, we used this constraint, together with the RoseTTAFold prediction algorithm (28), to generate a three-dimensional model of APCDD1. Although the resulting model was a relatively poor match to the final structure (Fig. 1*B*), it correctly captured the paired β-barrel domain structure of APCDD1 (Fig. 1*C*; described below). Fitting this model to the electron density map of eMBP-APCDD1 provided the starting point for iteratively solving the structure of the APCDD1 half of the fusion protein (Fig. 1 *D*–*F* and *SI Appendix*, Table S1). Molecular replacement with this structure was then used to solve the structure of APCDD1 in the two crystal forms described above (Fig. 1 *G* and *H*).

### Architecture of the APCDD1 ECD.

**Fig. 2.**
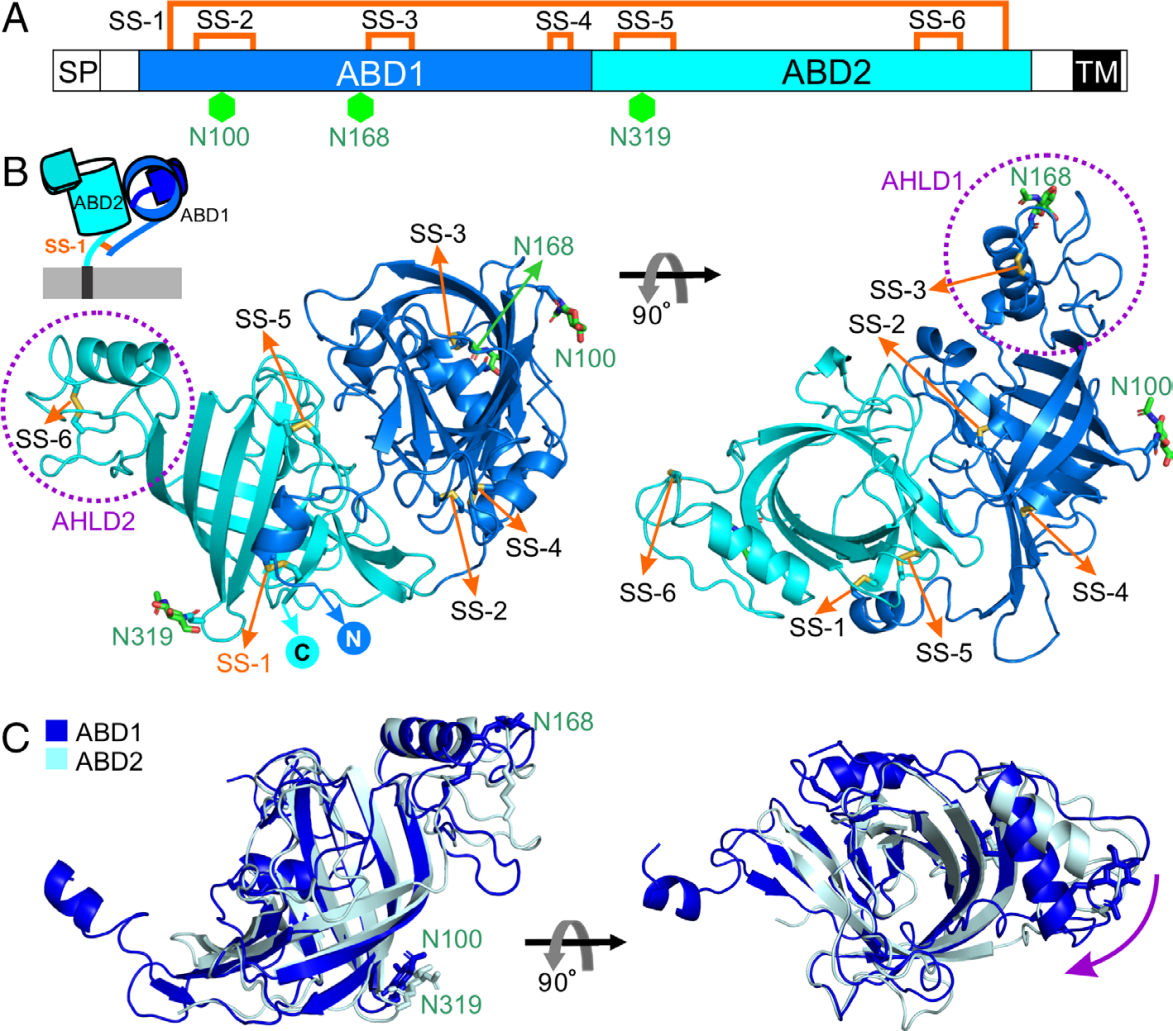
Structure of the APCDD1 ECD in the apo-form. (*A*) Schematic diagram of APCDD1 (SP, signal peptide; TM, transmembrane domain). ABD1 and ABD2 are colored in blue and cyan, respectively. Six disulfide bonds and three N-linked glycosylation sites are denoted as orange lines and green hexagons, respectively. (*B*) Ribbon representation of the ECD of APCDD1 (ABD1, blue; ABD2, cyan) in two views. AHLD1 and AHLD2 are marked with purple dotted circles. Disulfide bonds and N-linked glycans are shown as sticks. The N- and C-termini are labeled. The top-left *Inset* shows a cartoon of APCDD1 on the cell surface. The ABD1 and ABD2 β-barrels differ in orientation by approximately 90° and have an interdomain disulfide bond (SS-1). (*C*) Superposition of ABD1 and ABD2 (with rmsd of 2.11 Å over 160 Cα atoms) reveals high structural similarity between the β-barrels and an orientation difference between AHLD1 and AHLD2, as shown by the purple arrow.

**Fig. 3.**
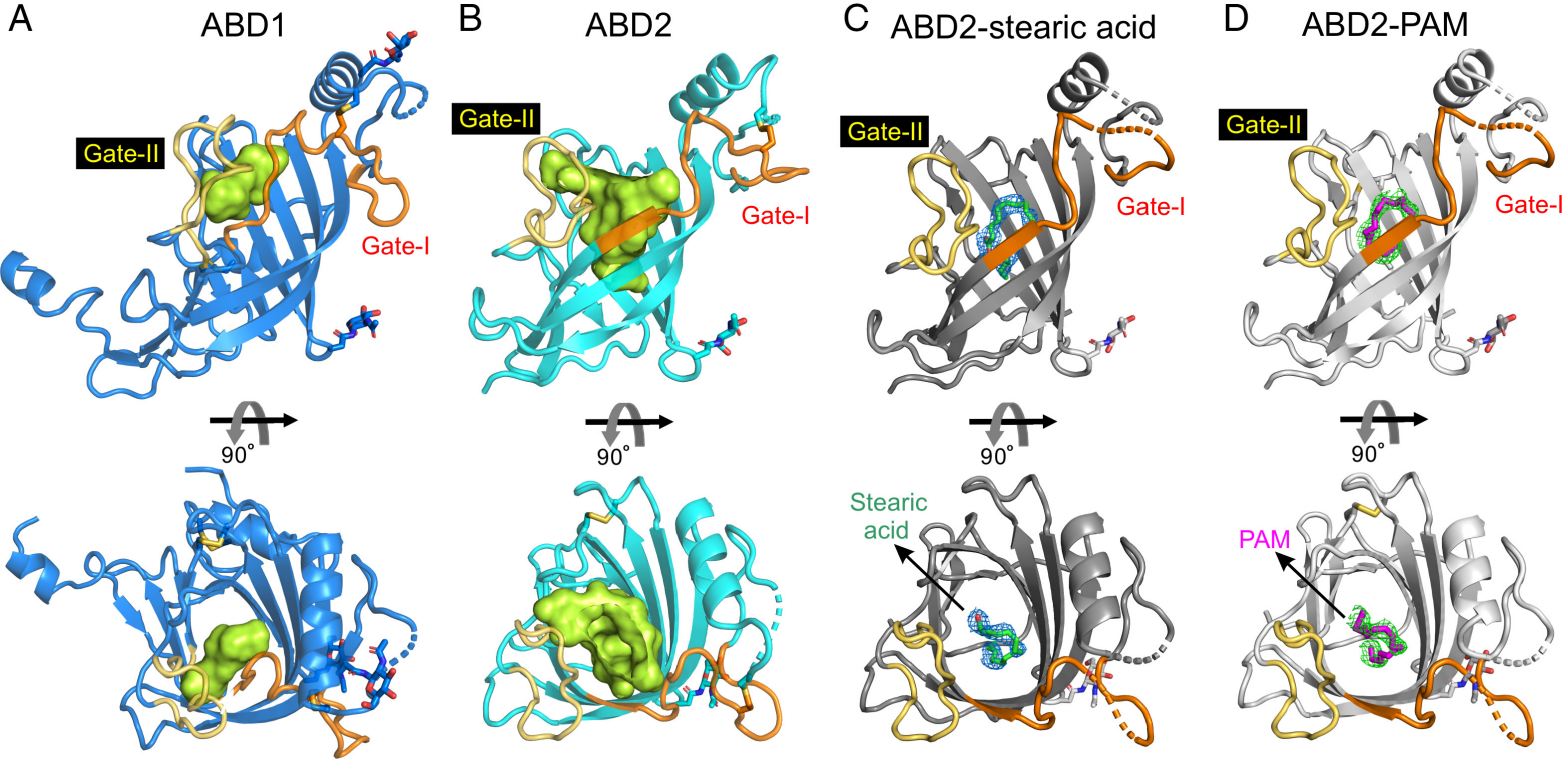
Structural analyses of ABD1, ABD2, and ABD2 in complex with a lipid. (*A*) Ribbon diagram of ABD1 (blue). The interior volume of the ABD1 pocket was rendered with CASTp (29) using a 1.4 Å probe and is colored green. The regions involved in determining pocket size are colored orange (Gate-I) and yellow (Gate-II). N-linked glycans are shown as sticks. (*B*) The structure of ABD2 (cyan) reveals a large hydrophobic pocket for ligand binding, rendered as described for (*A*) and is shown with a green interior volume. (*C*) Structure of ABD2 (eMBP-APCDD1 chain A; charcoal gray) was refined with stearic acid (green sticks) bound. The 2|*F*_O_|-|*F*_C_| electron density for stearic acid (blue meshes) is contoured at 0.9 σ. (*D*) Structure of ABD2 (eMBP-APCDD1 chain A; gray) was refined with PAM (magenta sticks) bound. The 2|*F*_O_|-|*F*_C_| electron density for PAM (green meshes) is contoured at 0.9 σ.

The structure of APCDD1 reveals an unusual architecture consisting of two β-barrel domains (ABD1 and ABD2) that differ in orientation by ~90° (Fig. 2 and *SI Appendix*, Fig. S2). ABD1 and ABD2 have, respectively, three and two intradomain disulfide bonds, a large area of interfacial contact, and one interdomain disulfide bond (Cys52–Cys466). This structure is observed in all three crystal forms (eight unique APCDD1 structures), with a global rmsd of 0.7 to 1.1 Å among structures (*SI Appendix*, Fig. S2*E* and Table S2). ABD1 and ABD2 present highly similar folds, consisting of two curved antiparallel β-sheets connected by an α-helix and loop domain (AHLD) (Fig. 2 *B* and *C*). In all three crystal forms, AHLD1 and AHLD2 differ by ~30° in orientation relative to their linked β-barrel domains (Fig. 2*C* and *SI Appendix*, Fig. S2 *E* and *F*). For both ABD1 and ABD2, the interior surface of the β-barrel is lined by hydrophobic amino acids. The barrels are open to the solvent on the side adjacent to AHLD, whereas the other side is closed by tight packing between side chains from β1 and β2 for ABD1 and from α3 and β12 for ABD2 (Fig. 3 *A* and *B*).

### Hydrophobic Pockets and Lipid Binding.

**Fig. 4.**
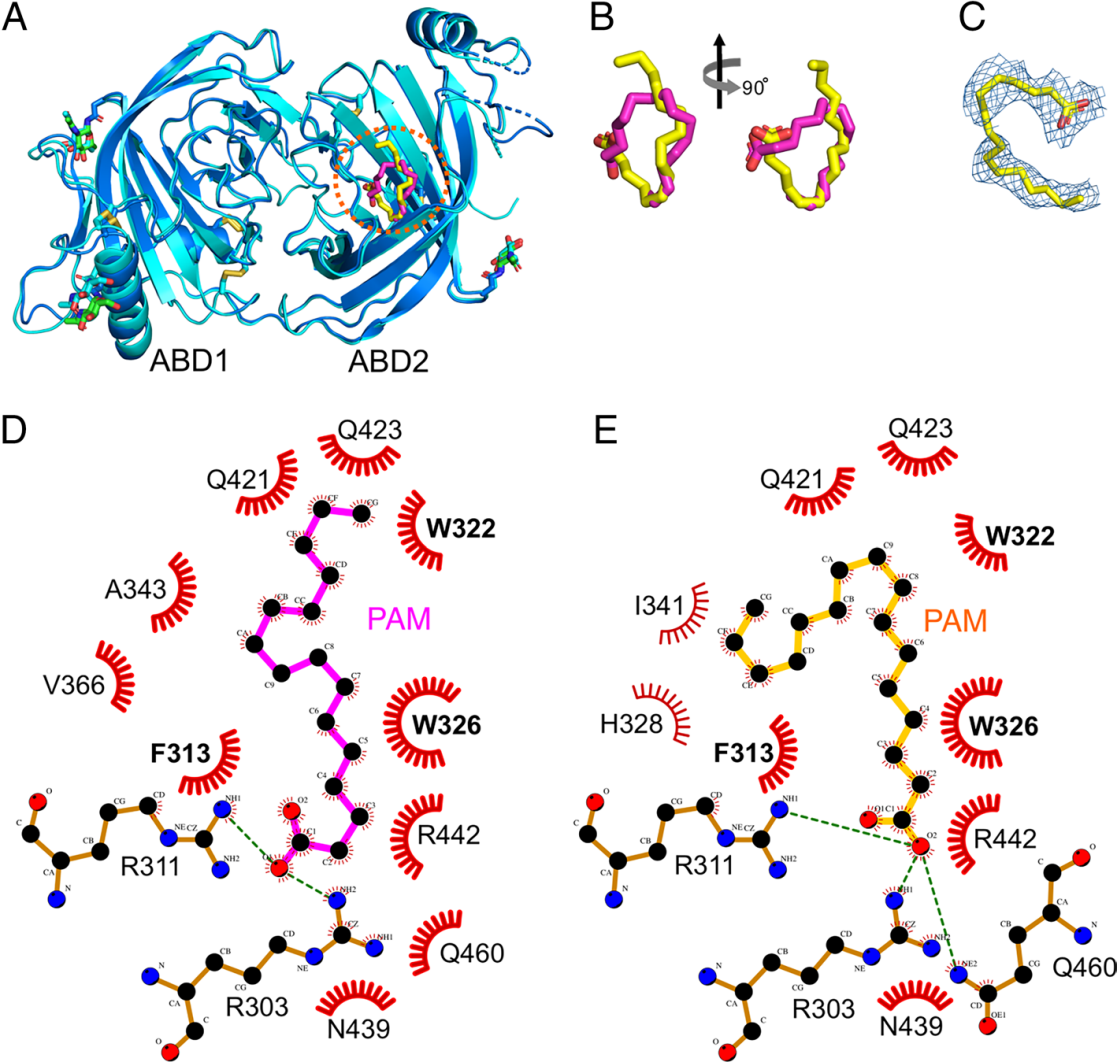
Lipid-binding pocket of ABD2. (*A*) Structural superposition of APCDD1 with bound PAM (magenta sticks) from eMBP-APCDD1 chain A and APCDD1 with bound PAM (yellow sticks) from eMBP-APCDD1 chain C. No major conformational changes are observed. The PAM-binding pocket of ABD2 is marked with a red dotted circle. (*B*) A close-up view of the PAM molecules as shown in (*A*). (*C*) The 2|*F*_O_|-|*F*_C_| electron density (blue meshes) of PAM (yellow sticks) from eMBP-APCDD1 chain C contoured at the 0.8 σ level. (*D* and *E*) Diagrams of ABD2 and PAM interactions generated with LigPlot+ (30). Panel (*D*), eMBP-APCDD1 chain A. Panel (*E*), eMBP-APCDD1 chain C. Atoms are colored as follows: nitrogen, blue; oxygen, red; carbon, black. Hydrogen bonds are displayed as green dashed lines. Red eyelashes denote hydrophobic interactions.

Intriguingly, we observed an electron density consistent with the acyl chain of a lipid molecule in the hydrophobic pocket of ABD2 in two of the four eMBP-APCDD1 structures (chains A and C; Figs. 3 *C* and *D* and 4*A*). We suspected that these lipid molecules became bound following APCDD1 secretion from HEK293 cells, which were grown in the presence of 2% bovine serum, a plentiful source of lipids. To define the identities of the bound molecules, we extracted hydrophobic molecules from the purified APCDD1 sample used for crystallization and analyzed the extracted species by high-performance liquid chromatography (HPLC) coupled with mass spectrometry (MS). This analysis revealed stearic acid, a C18 fatty acid (i.e., with 18 carbon atoms), as the most prevalent species (*SI Appendix*, Fig. S3 *A* and *B*). Therefore, we modeled a molecule of stearic acid into the electron density in the ABD2 pocket; the subsequent structural refinement revealed a good fit between stearic acid and the electron density (Fig. 3*C* and *SI Appendix*, Fig. S3 *C* and *D*).

Since APCDD1 inhibits WNT signaling and WNTs have a covalently linked palmitoleate (PAM; C16 fatty acid; ref. 1) that contributes to FRIZZLED binding and signaling (31–33), we also fitted a PAM molecule into the electron density in the ABD2 pocket (Figs. 3*D* and 4 *A*–*C*). The subsequent refinement showed a good fit between PAM and the electron density. Interestingly, this structural analysis also revealed different lipid conformations in the two APCDD1 structures (Fig. 4*B*), both of which exhibit multiple contacts between the lipid and hydrophobic side chains lining the ABD2 pocket (Fig. 4 *D* and *E*). A comparison between the bound and unbound APCDD1 structures shows no major conformational change upon lipid binding (*SI Appendix*, Fig. S2*F*).

**Fig. 5.**
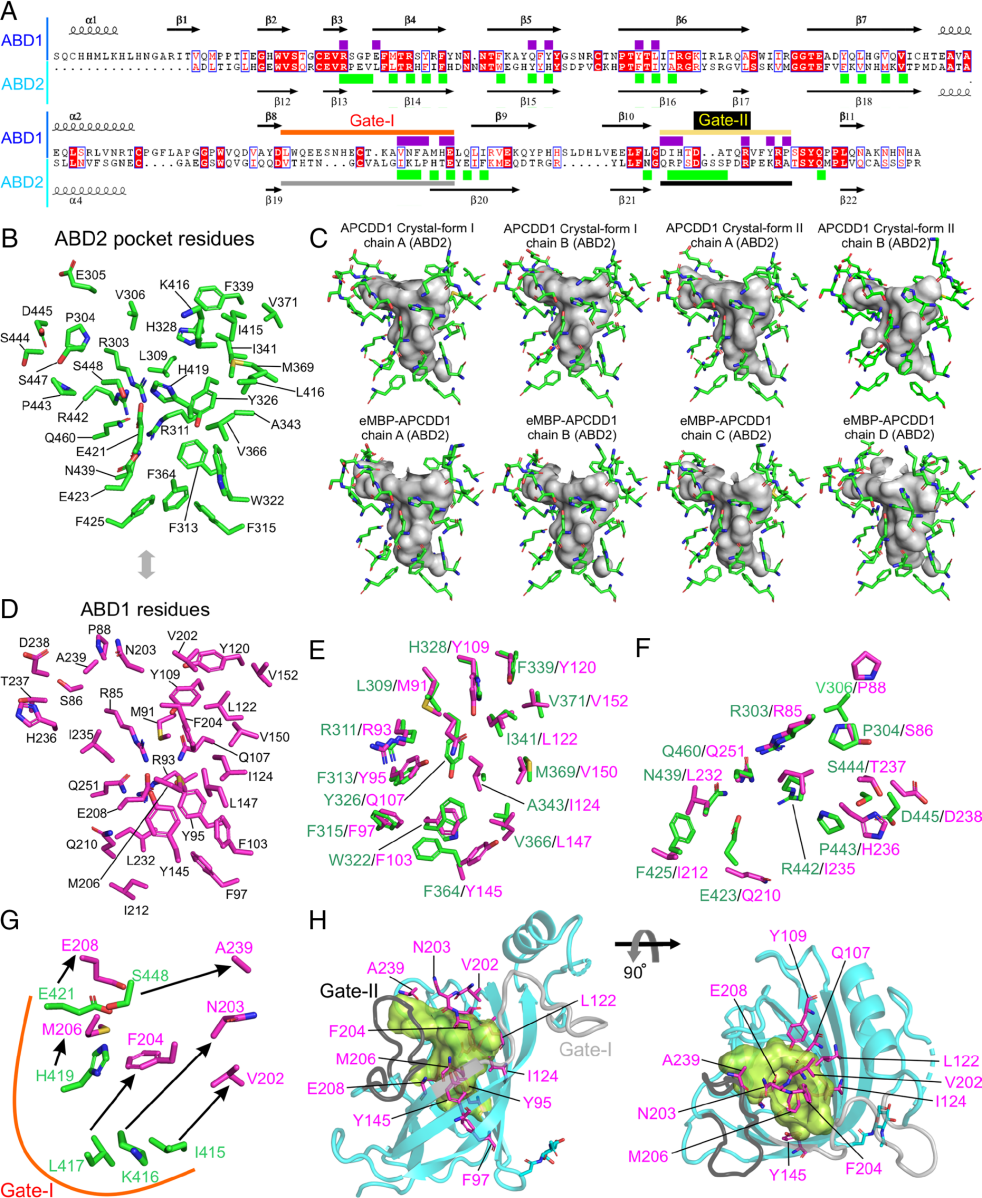
Structural analysis and comparison of hydrophobic pockets of ABD1 and ABD2. (*A*) Structure-based sequence alignment of ABD1 with ABD2. Secondary structure elements are represented. Purple and green rectangles highlight residues lining the hydrophobic pockets of ABD1 and ABD2, respectively. The regions of Gate-I and Gate-II are marked with horizontal colored lines. (*B*) Residues lining the hydrophobic pocket of ABD2 are shown as sticks and labeled. (*C*) The interior volume of the ABD2 pocket (gray), rendered with CASTp ((29)) together with residues that line it, are shown as green sticks, for the eight APCDD1 structures determined in this study. (*D*) Stick representation of ABD1 residues corresponding to the residues that line the ABD2 hydrophobic pocket. (*E*–*G*) ABD1 and ABD2 were superimposed (Fig. 3*A* and *B*) and subsets of the corresponding pairs of ABD1 and ABD2 residues were visualized as sticks (magenta for ABD1 and green for ABD2): (*E*) pairs with high spatial similarity; (*F*) pairs with moderate spatial similarity; (*G*) pairs with low spatial similarity (connected by arrows). (*H*) ABD2 ribbon diagram (cyan; with Gate-I and Gate-II colored light and dark gray, respectively) and the hydrophobic pocket rendered as a green interior volume. Select ABD1 residues that occupy or partially occupy the ABD2 hydrophobic pocket are visualized as magenta sticks with their positions and orientations determined by the superposition of ABD1 and ABD2.

**Fig. 6.**
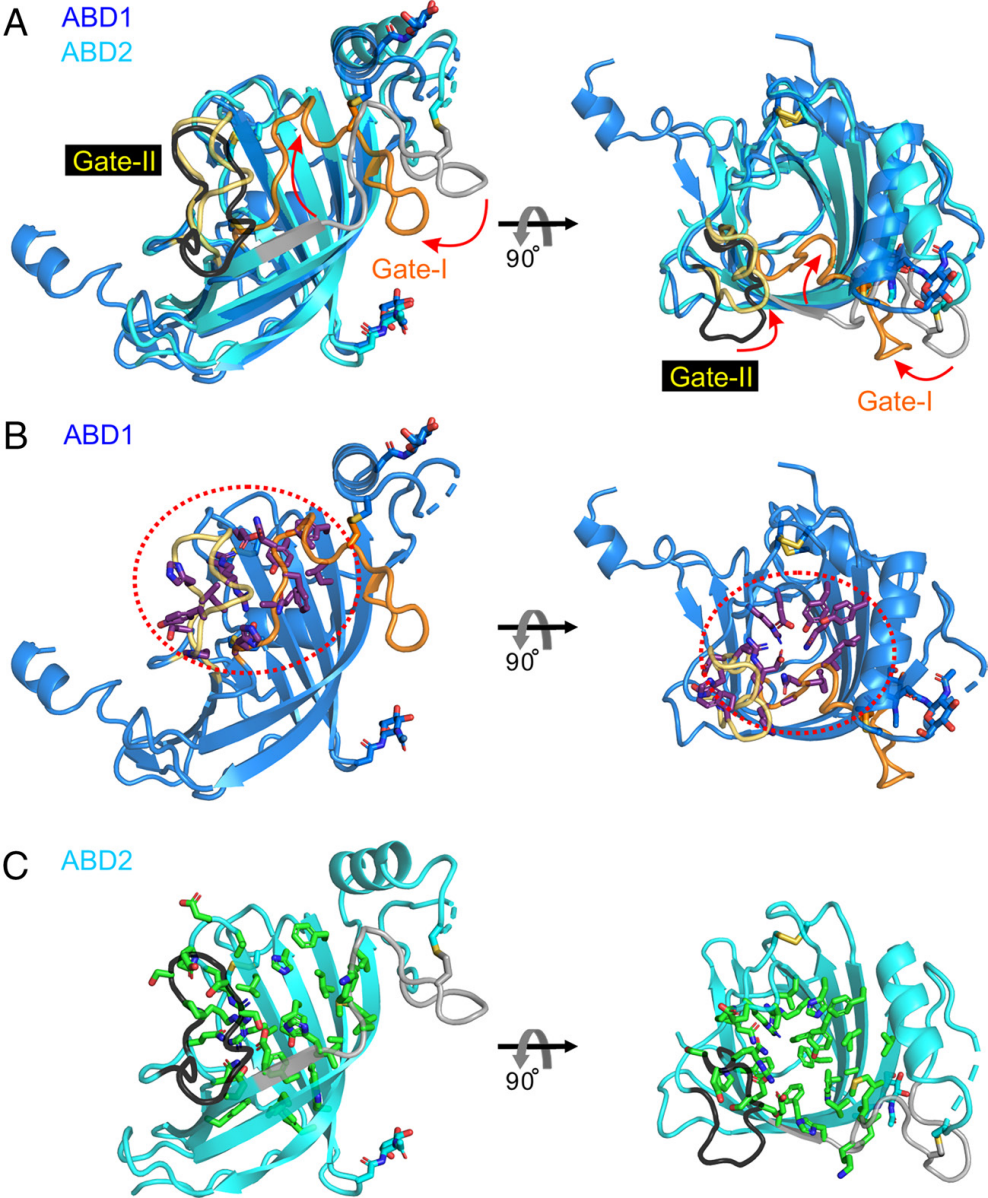
Structural comparison of ABD1 with ABD2. (*A*) Comparison between the structures of ABD1 (blue) and ABD2 (cyan). Gate-I (orange for ABD1 and gray for ABD2) and Gate-II (yellow for ABD1 and black for ABD2) are the two most important regions for determining pocket size. Red arrows indicate the change in these gates in comparing the ABD2 (large pocket) to ABD1 (small pocket) configurations. (*B*) Residues for the ABD1 pocket highlighted in Fig. 5*A* are shown as sticks (atom coloring: purple, carbon; blue, nitrogen; red, oxygen; yellow, sulfur). The red dotted circle highlights the region of the ABD1 pocket. (*C*) Residues for the ABD2 pocket highlighted in Fig. 5*A* are shown as sticks (atom coloring: green, carbon; blue, nitrogen; red, oxygen; yellow, sulfur).

Despite the nearly identical backbone configurations of the β-barrel residues in ABD1 and ABD2, the interior volumes of their hydrophobic pockets are dramatically different: ~35 Å^3^ for ABD1 vs. ~350 Å^3^ for ABD2 (Fig. 3 *A* and *B* and *SI Appendix*, Table S3), as calculated with CASTp (29). To define the molecular basis for this difference, we superimposed the structures of ABD1 and ABD2 (Figs. 2, 5, and 6). Two loops, which we refer to as Gate-I and Gate-II, differ markedly in both primary sequence and configuration between ABD1 and ABD2 (Figs. 3 *A* and *B*, 5, and 6). In ABD1, Gate-I and Gate-II are positioned closer to the center of the β-barrel, whereas, in ABD2, Gate-I and Gate-II are positioned away from the center of the β-barrel (Figs. 3 *A* and *B* and 6). The differences between ABD1 and ABD2 can be further appreciated by comparing the identities and spatial locations of the individual amino acid side chains that line their hydrophobic pockets (Fig. 5 *B*–*D*). In particular, Gate-1 side chains show a concerted inward shift within the ABD1 pocket and, together with multiple other side chains in ABD1, largely occupy the interior of the superimposed ABD2 pocket (Fig. 5 *E*–*H*). These distinguishing features of the ABD1 and ABD2 pockets are observed in all eight APCDD1 structures (Fig. 5*C* and *SI Appendix*, Table S3).

With respect to the function of the individual ABD1 and ABD2 domains, our attempts to address this question by producing the individual domains have thus far failed, most likely due to the instability of the individual domains. We note that the two domains have a large area of interdomain contact and an interdomain disulfide bond, which likely stabilizes them.

### Comparisons between APCDD1 and Other Lipid-Binding Proteins.

**Fig. 7.**
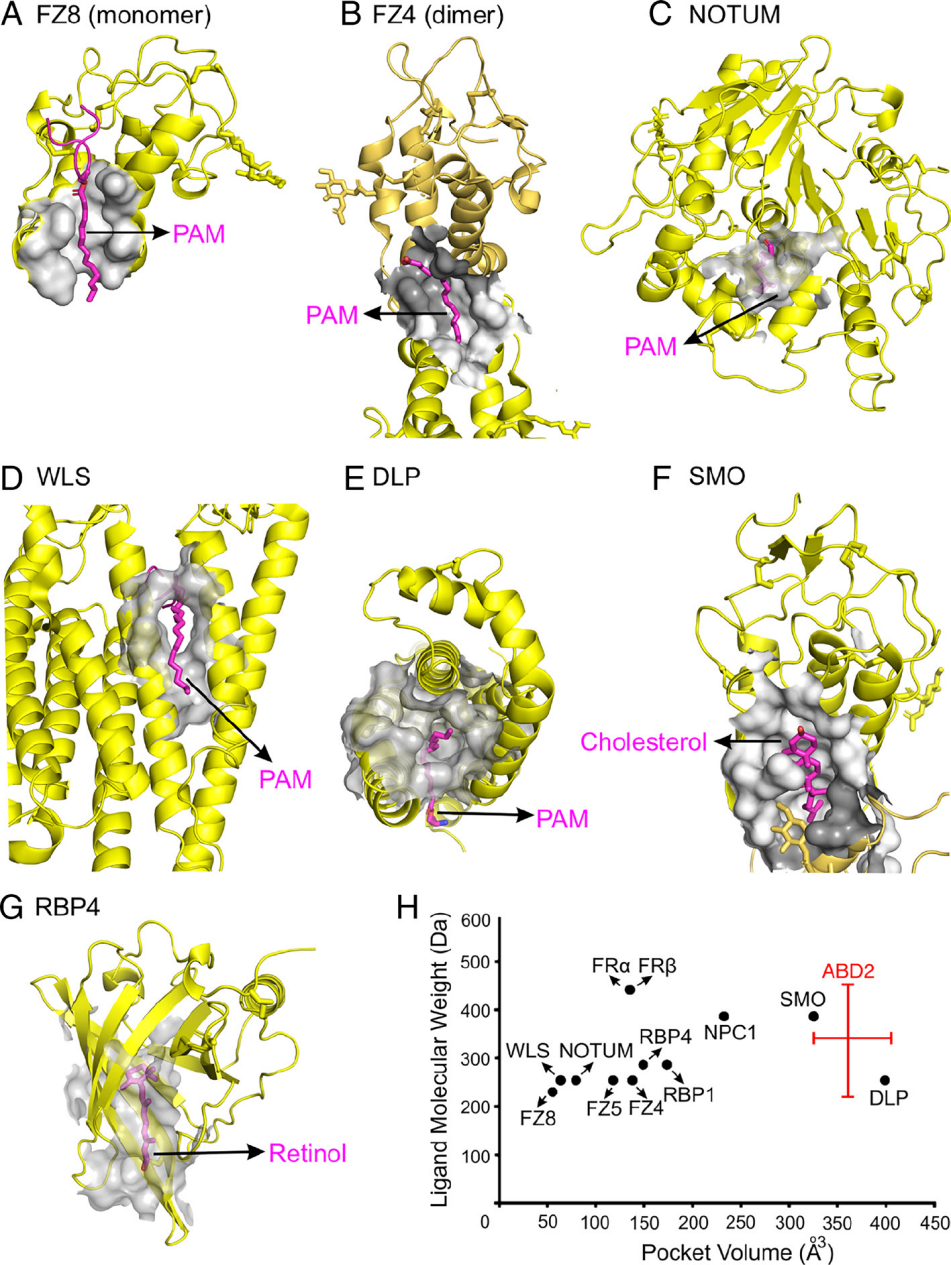
Structural analyses of hydrophobic ligand-binding pockets of cell surface and secreted proteins. Hydrophobic ligand volumes (gray) and hydrophobic ligands (magenta sticks), rendered with CASTp, are shown for the following lipid-binding pockets. (*A*) FZD8 CRD in complex with the PAM of WNT8A (PDB ID 4F0A). (*B*) FZD4 CRD dimer interface in complex with PAM (PDB ID 5UWG). (*C*) NOTUM in complex with the PAM of a WNT7A peptide (PDB ID 4UZQ). (*D*) WLS in complex with the PAM of WNT8A (PDB ID 7KC4). (*E*) DLP in complex with the PAM of a WNT7A peptide (PDB ID 6XTZ). (*F*) SMOOTHENED (SMO) in complex with cholesterol (PDB ID 5L7D). (*G*) Retinol binding protein 4 (RBP4) in complex with retinol (PDB ID 1RBP). (*H*) Plot of the interior volume of the hydrophobic ligand-binding pocket in the indicated proteins vs. the molecular weight of their cognate hydrophobic ligands. The range of values for the ABD2 hydrophobic pocket volume (horizontal red bar) reflects differences among the eight APCDD1 structures and the vertical bar estimates the range of potential ligand molecular weights predicted from the volume. Quantifications are in *SI Appendix*, Table S3.

Binding to the PAM that is covalently linked to WNT ligands is a recurrent theme among protein that transduce or modulate WNT signals. These proteins include FRIZZLED (33), NOTUM (7, 8), WNTLESS (WLS) (34), and DALLY-LIKE (DLP) (35). A comparison of the structures of these and other proteins in complex with hydrophobic ligands shows great diversity in the volumes of their lipid binding cavities, ranging from ~55 Å^3^ to ~400 Å^3^ (Fig. 7 *A*–*G* and *SI Appendix*, Fig. S4 and Table S3). At ~350 Å^3^, the ABD2 pocket is at the high end of this distribution and is predicted to accommodate hydrophobic ligands in the 230 to 440 Da range (Fig. 7*H* and *SI Appendix*, Table S3). The shallow ABD1 pocket might accommodate a small ligand or part of a larger ligand.

To explore the evolutionary origin of APCDD1, we performed a 3D structure search of the Protein Data Bank (PDB) and AlphaFold (36) databases using the DALI server (37). This search returned weak homologs, including the lipocalin and peripheral myelin protein 2 (P2) families, with primary sequence identities in the 5 to 10% range. As seen for ABD1 and ABD2, the members of these protein families have a hydrophobic binding pocket formed by a β-barrel domain (*SI Appendix*, Fig. S5). Detailed structural comparisons between APCDD1 and members of the lipocalin and P2 families showed marked differences in the shapes of their β-barrels and the orientations of β-sheets, giving an average rmsd of ~2.4 to 3.7Å (*SI Appendix*, Fig. S5). Proteins in the lipocalin and P2 families bind a wide variety of hydrophobic molecules via the pocket within their β-barrel domains (38). Across both families, the mean volume of the hydrophobic pocket is ~267 Å^3^, as calculated with CASTp (29).

### Evolution of APCDD1 and APCDD1L.

Nearly all vertebrate genomes code for two APCDD1 homologous sequences: APCDD1 and APCDD1-like (APCDD1L), which exhibit ~50% amino acid identity throughout their length (*SI Appendix*, Fig. S6). The APCDD1L sequences retain the principal features of the APCDD1 sequence, including the 12 conserved and disulfide-bonded cysteines (*SI Appendix*, Fig. S6). Interestingly, APCDD1L amino acid sequences show a several-fold greater rate of evolutionary change compared to APCDD1 sequences, as seen in the dendrogram in *SI Appendix*, Fig. S7*A*. Threading the rat APCDD1L sequence into the mouse APCDD1 structure suggests that APCDD1L adopts very nearly the same structure, with an ABD2 pocket that can also accommodate a lipid (*SI Appendix*, Fig. S7). At present, the function of APCDD1L is unknown.

### APCDD1 Binds to WNT7A.

Based on the structure of APCDD1, the most attractive hypothesis for its action as a WNT inhibitor is that it binds directly to WNTs via their covalently linked PAM. To test this idea, we chose WNT7A from among the 19 mammalian WNTs, because WNT7A, like APCDD1, regulates CNS vascular development and barrier maturation (39). More specifically, WNT7A produced by glia and neurons activates FRIZZLED receptors on CNS endothelial cells, which respond with high-level WNT signaling, including high-level *Apcdd1* expression.

**Fig. 8.**
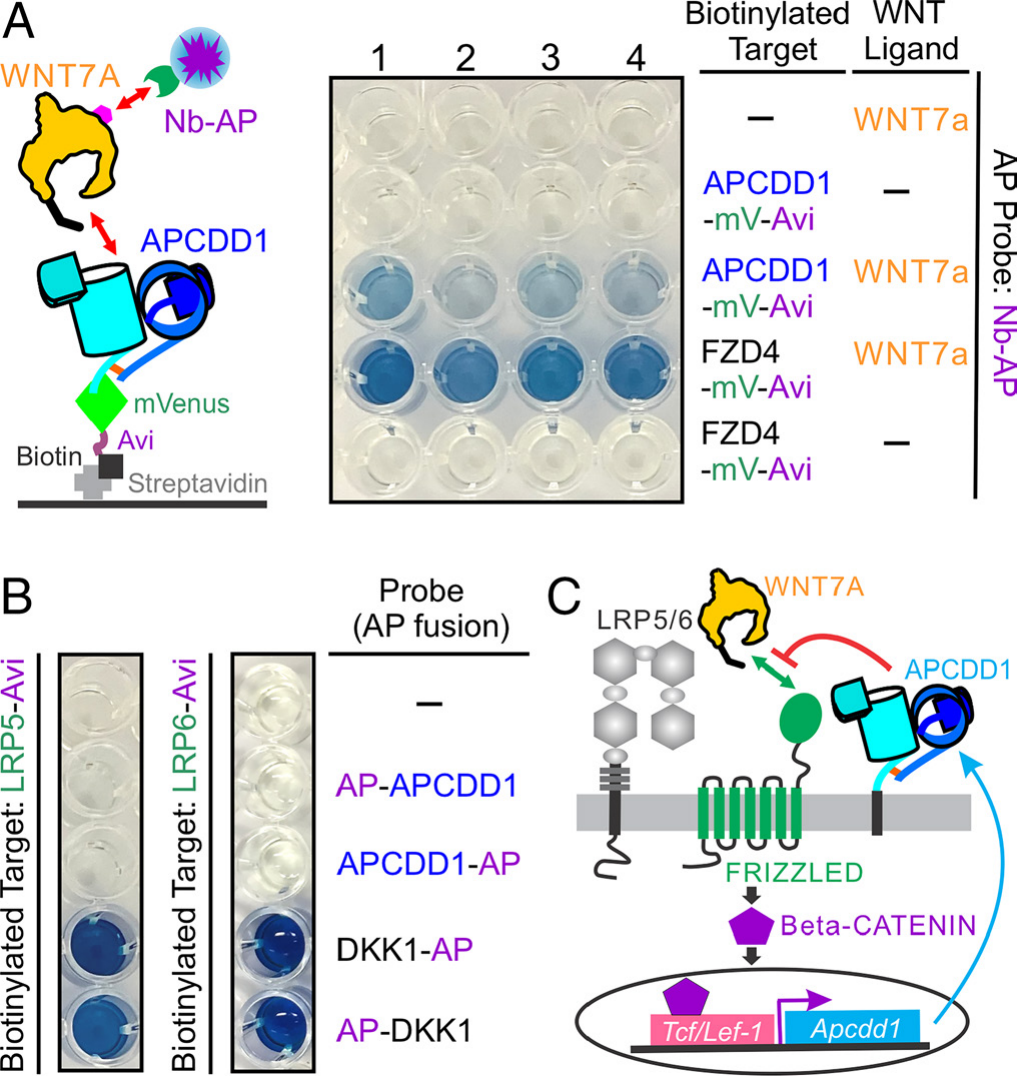
Functional characterization of APCDD1. (*A*) APCDD1 binds to WNT7A. *Left*, diagram of the protein–protein interaction assay used in this study. The secreted complex between the APCDD1-mVenus-His-tag fusion protein with biotinylated Avi-tag at its C-terminus and WNT7A-His tag with an ALFA-tag at its C terminus (magenta hexagon) was concentrated by IMAC affinity chromatography and then immobilized on streptavidin-coated wells. Captured WNT7A was detected using an anti-ALFA nanobody (Nb)-AP fusion protein and a colorimetric AP reaction. Right, WNT7A binding to APCDD1 and the FZD4 CRD. The binding and washing were conducted in the following buffer conditions: 1, standard buffer; 2, in the presence of 1% DDM; 3, in the presence of 0.1% DDM; 4, in the presence of 2.25% Triton X-114 (*Materials and Methods*). The FZD4 CRD bait serves as a positive control. (*B*) APCDD1 does not detectably bind to the extracellular PE1-4 domains of LRP5 or LRP6 as determined by probing with APCDD1-AP and AP-APCDD1. The DKK1-AP and AP-DKK1 probes serve as positive controls. (*C*) Model showing how *Apcdd1* expression and APCDD1 function provide negative regulatory feedback in canonical WNT signaling.

Most WNTs, including WNT7A, are not well behaved biochemically and they typically form inactive aggregates following secretion into conditioned medium (40). However, in some instances, coexpression and coassembly with a binding partner facilitates secretion of a native WNT complex (33, 40, 41). Therefore, we decided to coexpress in HEK293T cells ALFA epitope-tagged WNT7A with either a C-terminal biotinylated APCDD1-mVenus fusion or, as a positive control, a C-terminal biotinylated FZD4 cysteine-rich domain (CRD)-mVenus fusion. We then applied immobilized metal affinity chromatography (IMAC) to concentrate the preformed protein complexes from the conditioned medium, using the histidine tags on the epitope-tagged WNT7A and the mVenus fusions of APCDD1 and FZD4 CRD. The partially purified complexes were captured on streptavidin-coated wells, and the epitope-tagged WNT7A protein was detected with an anti-ALFA nanobody-alkaline phosphatase (AP) fusion protein and a colorimetric AP assay (Fig. 8*A*). The FZD4 CRD showed a strong WNT7A binding signal and APCDD1 showed a weaker WNT7A binding signal. Omitting either the biotinylated bait or the WNT7A ligand eliminated the binding signal.

To address the hypothesis that the PAM linked to WNT7A binds to the hydrophobic pocket in APCDD1, we compared the WNT7A capture efficiency in the presence vs. the absence of neutral detergents [dodecyl maltoside (DDM) and Triton X-114], which would be expected to both compete for occupancy of the lipid-binding pocket and stabilize the unbound PAM. Both detergents reduced the WNT7A-APCDD1 binding signal, with 1% DDM showing a greater potency than 0.1% DDM (Fig. 8*A*). In contrast, the same detergent treatment had little effect on the interaction of WNT7A and FZD4 CRD (Fig. 8*A*), most likely because this interaction is stabilized by both protein–lipid and protein–protein contacts and because the PAM-binding pocket in the FZD CRD consists of a narrow groove that makes a tight fit to the PAM (33, 41). Although a definitive analysis of the WNT–APCDD1 interaction will require a high-resolution structure of the complex, these biochemical data are consistent with a model in which the PAM moiety of WNT binds to the hydrophobic pocket in APCDD1.

To determine whether APCDD1 directly interacts with the extracellular four tandem β-propeller-epidermal growth factor-like domain pairs (PE1-4) of LRP5/6 coreceptors, we produced PE1-4 of LRP5 and LRP6 with a C-terminal biotin tag, captured them on streptavidin-coated wells, and probed the wells with APCDD1-AP and AP-APCDD1 fusion proteins or, as a positive control, with DKK1-AP and AP-DKK1 fusion proteins (Fig. 8*B*). We observed no detectable binding between the APCDD1 and the PE1-4 domains of LRP5 or LRP6, consistent with a model in which APCDD1 acts via binding to WNT ligands rather than to WNT coreceptors.

The lower WNT7A-APCDD1 binding signal compared to the WNT7A-FZD4 CRD binding signal presumably reflects a lower affinity for the former interaction. If this differential binding also applies in vivo, it may be partially offset by the greater abundance of transcripts coding for APCDD1 relative to transcripts coding for WNT receptors and coreceptors. For example, in mouse brain vascular endothelial cells, in which a high level of canonical WNT signaling maintains the blood–brain barrier, *Apcdd1* transcripts are 5 to 100 fold more abundant than transcripts coding for WNT receptor (FZD4), coreceptor (LRP5 and LRP6), and coactivator (GPR124 and RECK) proteins (*SI Appendix*, Fig. S8). Although the corresponding protein abundances in vivo are not presently known, the relative transcript abundances suggest that in brain vascular endothelial cells APCDD1 may be substantially more abundant than WNT receptors, coreceptors, and coactivators.

In sum, the experiments described here reveal APCDD1 to be a transmembrane lipid-binding protein, and they suggest that APCDD1 exerts its inhibitory effect on WNT signaling by binding to lipidated WNTs (Fig. 8*C*).

## Discussion

The present study shows that the ECD of APCDD1 consists of two homologous β-barrel domains (ABD1 and ABD2), linked by one interdomain disulfide bond. Protein sequence searches and protein fold comparisons show that APCDD1 exhibits an unusual architecture. Interestingly, structural analyses show that ABD2, but not ABD1, has a hydrophobic pocket that is larger than the hydrophobic binding pockets in FRIZZLED (33), NOTUM (7, 8), and WLS (34), each of which binds the PAM that is covalently linked to WNT ligands. Evidence that ABD2 could plausibly bind the WNT-linked PAM comes from our observations of a) electron density consistent with a C16 or C18 lipid in the hydrophobic pocket of ABD2 in two of four eMBP-APCDD1 structures, and b) copurification of stearic acid with APCDD1. In support of this model, in vitro binding assays show that APCDD1 binds to WNT7A. Building on earlier reports that APCDD1 inhibits WNT signaling in transfected cells (17–19), the present study suggests that APCDD1 serves as a negative feedback regulator by titrating WNT ligands at the cell surface to reduce WNT signaling. At a technical level, this work also demonstrates the synergy that is possible between artificial intelligence-based structure prediction algorithms and traditional molecular replacement using fusion-partner-derived phase information for protein structure determination.

The PAM that is covalently joined to WNT proteins plays a central role in WNT-CRD binding (31, 33, 41–44), secretion dependent upon WNT association with WLS (34), the formation of a WNT morphogen gradient via interaction with DLP (35), and WNT signaling inhibition by NOTUM, a deacylase that recognizes palmitoylated WNT and releases the PAM moiety (7, 8). As shown in Fig. 7 and *SI Appendix*, Fig. S4, the hydrophobic PAM-binding pockets in these WNT-interacting proteins exhibit substantial shape and size diversity. In the FRIZZLED CRD monomer and dimer-binding modes, and in WLS and DLP, the PAM-binding pockets are extended and narrow, whereas NOTUM contains a compact and globular hydrophobic PAM-binding pocket. By contrast, the structures of APCDD1 determined here reveal that the ABD2 pocket has a large, open, and globular shape that is predicted to accommodate hydrophobic ligands with molecular masses in the 230 to 440 Da range, including PAM (254 Da). Determining whether the APCDD1–WNT interaction also involves protein–protein interactions will likely require a high-resolution structure of the complex.

Genome sequences predict the existence of APCDD1 homologues in nearly all vertebrates, as well as in a wide variety of invertebrates, including many evolutionarily distant Metazoa. Among the 100 most similar invertebrate APCDD1 homologues, amino acid alignments with mammalian APCDD1 show 25 to 40% amino acid identity spread across the entire ECD. The distinction between APCDD1 and APCDD1L is clear in comparing vertebrate homologues (*SI Appendix*, Fig. S7*A*), but many invertebrate genomes carry one or more APCDD1/APCDD1L sequences with approximately the same degree of similarity to APCDD1 and APCDD1L. Since WNT signaling arose early in metazoan evolution (45), it is possible that the earliest APCDD1/APCDD1L homologues evolved as WNT regulators and that they maintain this function in present day Metazoa.

In addition to the mouse *Apcdd1* knockout and overexpression phenotypes described in the Introduction, *Xenopus* embryo experiments with *Apcdd1* morpholino oligonucleotide knockdown reveal expansion in the expression domain of the ventral marker *Sizzled* and a reduction in the expression of the dorsal (neural tube) marker *Sox2* (46). *Apcdd1l* morpholino oligonucleotide knockdown and TALEN-mediated germ-line elimination of *Apcdd1l* in zebrafish show that a) homozygous loss of *Apcdd1l* is dispensable for viability and fertility, and b) progeny embryos from *Apcdd1l* homozygous null parents, which lack both maternal and zygotic *Apcdd1l* function, show expansion of the Spemann organizer region (visualized by the expression of *gsc*), a phenotype that appears to be of little consequence for subsequent development (46). The *Apcdd1* and *Apcdd1l* loss-of-function phenotypes are consistent with enhanced WNT signaling, and they further suggest that APCDD1 may additionally inhibit Bone Morphogenetic Protein (BMP) signaling (46). It will be interesting to further explore the BMP inhibitor hypothesis and to determine whether combined elimination of both *Apcdd1* and *Apcdd1l* in mice, frogs, or fish produces a distinctive and/or more severe developmental phenotype than elimination of either gene alone.

The structure of APCDD1 presents a distinctive protein architecture with ABD1 and ABD2 closely packed in an orientation that differs by ~90°, with one interdomain disulfide bond (Fig. 2). In comparisons to the lipocalin and P2 families, the ABD1 and ABD2 β-barrel domains show 5 to 10% amino acid sequence identity and relatively weak structural homology. The lipocalin/P2 superfamily predates APCDD1, with superfamily members present in genome sequences in all domains of life except for Archaea. The members of the lipocalin/P2 superfamily are highly divergent at the primary sequence level and are well known for their roles in binding and transporting hydrophobic molecules (e.g., lipids, pheromones, steroid hormones, and retinoids) using the hydrophobic pocket within the β-barrel domain (38). As a distant relative of this family, APCDD1 is unusual in having a membrane anchor and two β-barrels in the same polypeptide. Interestingly, a secreted lipocalin family member in *Drosophila*, SWIM, has been reported to interact with WINGLESS (one of seven *Drosophila* WNTs) in a PAM-dependent manner to maintain WINGLESS solubility (47). Therefore, SWIM, like APCDD1, appears to use its hydrophobic pocket within the β-barrel domain for WNT binding via the WNT-linked PAM. It will be interesting to determine whether APCDD1 or SWIM exhibits any binding selectivity for specific WNTs.

A diverse collection of extracellular and intracellular proteins have been found to negatively regulate WNT signaling. sFRP and WIF1 prevent WNT and FRIZZLED interaction by capturing WNT ligands (2, 3); DKK, SOST, and KREMEN block the formation of the WNT–FRIZZLED-LRP complex and/or reduce the cell surface concentration of LRP5/LRP6 (4–6); NOTUM a WNT-specific deacylase, inhibits WNT signaling by removing the PAM from WNT ligands (7, 8); TIKI1, a transmembrane protease, suppresses WNT signaling by degrading WNT ligands (9); ZNRF3 and RNF43, transmembrane E3 ubiquitin ligases, ubiquitylate FRIZZLED to promote its degradation (10, 11); and AXIN2 intracellularly promotes β-CATENIN phosphorylation and degradation (12). APCDD1 represents a distinctive type of negative feedback regulator of WNT signaling. The APCDD1 structure presented here, together with the WNT-binding data, imply a mode of action in which APCDD1 titrates WNTs at the surface of responding cells to reduce WNT-FRIZZLED binding. Whether APCDD1 binding leads to internalization and/or degradation of WNTs and/or WNT-associated surface proteins remains to be determined. The upregulation of *Apcdd1* transcripts in response to WNT signaling in both normal and pathologic contexts implies that APCDD1 feedback effects are likely to be of broad biological relevance.

## Materials and Methods

### Sequence-Based and Phylogenetic Analyses.

The sequence-based homology search for the mouse APCDD1 sequence (UniProt code: Q3U128; ECD, residues 27 to 492) was carried out over the PDB database using the HHpred server (48). For the prediction of protein secondary structure and disordered regions, Ali2D (49–51) and Quick2D (52) were used.

For phylogenetic analysis, the amino acid sequences of APCDD1 and APCDD1L were aligned using ClustalΩ (53), and the phylogenetic tree was constructed with the neighbor-joining method using MEGA7 (54, 55). The optimal tree with the sum of branch lengths = 2.41744267 is shown. The phylogenetic tree is drawn to scale, with branch lengths in the same units as those of the evolutionary distances used to infer the tree. The evolutionary distances were computed using the Poisson correction method and are in the units of the number of amino acid substitutions per site (55).

### Plasmid Design and Construction.

For mouse APCDD1 constructs, a series of coding segments of the APCDD1 ECD were cloned into pHLsec-mVenus-12H (27, 56, 57) for expression in human embryonic kidney (HEK293) cells. For crystallization, the APCDD1 ECD (residues 27 to 481) was cloned into pHLsec-8H (58) with a C-terminal 8xHis tag. For the eMBP-APCDD1 construct, the APCDD1 ECD (residues 47 to 481) was cloned into pHLsec-eMBP-8H with an N-terminal eMBP fragment [PCR amplified from pET-11d-eMBP-8H (27)] and a C-terminal 8xHis tag.

For protein–protein interaction experiments, coding segments of the APCDD1 ECD and the human FZ4 CRD (56) were cloned into pHLsec-3C-mVenus-Avi-8H (57) with a C-terminal Human Rhinovirus-3C protease cleavage site followed by a monomeric (m)Venus fusion protein, an Avi tag and finally an 8xHis tag; the resulting plasmids were named APCDD1-mV-Avi and FZ4-mV-Avi, respectively. The mouse LRP5 PE1-4 construct (LRP6-Avi) and the human LRP6 PE1-4 construct (LRP6-Avi) were generated in pHL-Avitag3 with a C-terminal Avi tag as described previously (59). For AP fusion protein constructs, the APCDD1 ECD (residues 27 to 486) and human DKK1 (UniProt code: O94907; residues 32 to 266) were cloned into pHL-N-AP-Myc-8H (with an N-terminal human AP followed by a Myc tag and an 8xHis tag) and pHLsec-C-Myc-AP-8H (with a Myc tag followed by human AP and an 8xHis tag) (57); the resulting plasmids were named AP-APCDD1, APCDD1-AP, DKK1-AP, and AP-DKK1. Human WNT7A (UniProt code: O00755; residues 32 to 349) was cloned into pHLsec-ALFA-8H with an N-terminal ALFA sequence (SRLEEELRRRLTE) (60) and a C-terminal 8xHis tag. The nanobody against ALFA (NbALFA) (60) was cloned into pET-11d-C-Myc-eAP-8H (with a C-terminal Myc tag followed by *Escherichia coli* AP and finally an 8xHis tag, generating Nb-AP. All constructs were confirmed by DNA sequencing.

### Protein Expression and Purification.

HEK293T (ATCC CRL-11268) cells were maintained in a humidified 37 °C incubator with 5% CO_2_ in Dulbecco’s modified eagle medium (MilliporeSigma) supplemented with 2 mM L-Glutamine (L-Glu, Gibco), 0.1 mM nonessential amino acids (Gibco) and 10% [v/v] Fetal Bovine Serum (FBS, Gibco). The FBS concentration was lowered to 2% [v/v] after transfection with the DNA using polyethylenimine (PEI; MilliporeSigma) as described previously (61). For crystallization experiments, APCDD1 and eMBP-APCDD1 were expressed in HEK293T cells grown in HYPER*Flask*^®^ Cell Culture Vessels (Corning) and cultured in the presence of 5 μM of the class I α-mannosidase inhibitor, kifunensine (62) and 4 mM valproic acid (56) after transfection. Conditioned media were collected 5 d posttransfection and supplemented with 10 mM HEPES, pH 7.5, and 5 mM imidazole. The His-tagged sample was purified by IMAC using Ni Sepharose Excel resin (GE Healthcare Life Sciences). The IMAC eluted sample was subjected to size-exclusion chromatography using HiLoad Superdex 200 pg (GE Healthcare Life Sciences) in 10 mM Bis-Tris, pH 6.5, 0.3 M NaCl (for APCDD1) and 10 mM HEPES, pH 7.5, 0.15 M NaCl (for eMBP-APCDD1).

For *E. coli* expression and purification of Nb-AP, the plasmid DNA was transformed into *E. coli* BL21 Star™ (*DE3*) cells (ThermoFisher) and induced with 0.2 mM isopropyl β-thiogalactopyranoside in Luria broth containing 100 μg/mL ampicillin (MilliporeSigma) at room temperature (~25°C) overnight. The cell pellets were harvested by centrifugation and resuspended in B-PER bacterial protein extract reagent (ThermoFisher) supplemented with 50 mM HEPES, pH 7.5, 0.3 M NaCl, 30 mM imidazole, 1 mM MgCl_2_, 500 U benzonase (MilliporeSigma), 0.2 mg/mL lysozyme, and cOmplete Protease Inhibitor Cocktail (MilliporeSigma). The cell lysate was clarified by centrifugation, and the supernatant was filtered using a 0.45-μm Steritop filter (MilliporeSigma). Proteins were purified by IMAC using Ni Sepharose 6 Fast Flow resin (GE Healthcare Life Sciences). The eluted sample was dialyzed against 10 mM HEPES, pH 7.5, 0.15 M NaCl.

### Crystallization and Data Collection.

Purified APCDD1 protein was concentrated to 12.5 mg/mL in the presence of 0.5% *Flavobacterium meningosepticum* endoglycosidase-F1 (Endo-F1) prepared as described previously (56) for in situ deglycosylation (58). Purified eMBP-APCDD1 protein was concentrated to 11 mg/mL in the presence of 0.18 mM zinc acetate, 20 mM maltose, 0.5% Endo-F1, and 0.5% carboxypeptidase A/B (MilliporeSigma) for in situ deglycosylation and proteolysis (58). Using a Mosquito LCP crystallization robot (TTP Labtech), the protein samples were then subjected to sitting drop vapor diffusion crystallization trials in 96-well MRC 2 Well UVXPO plates (Hampton Research) by mixing 100 nL protein solution with 100 nL reservoir. APCDD1 crystal-form I crystallized in 0.7 M magnesium formate, 0.1 M Bis-Tris propane, pH 7.0. APCDD1 crystal-form II crystallized in 0.1 M magnesium acetate, 0.1 M sodium citrate, pH 5.8, 14% polyethylene glycol (PEG) 5K MME. Crystals of eMBP-APCDD1 were grown and optimized in 0.2 M ammonium citrate, pH 7.0, 20% PEG 3350, 4% NDSB-256, 5% glycerol.

For cryoprotection, crystals were transferred into a reservoir solution supplemented with 70% Tacsimate™, pH 7.0 for crystal-form I, with 20% glycerol for crystal-form II, and with a 5% increase gradually to 15% glycerol for eMBP-APCDD1, and subsequently cryocooled in liquid nitrogen. X-ray diffraction data were collected at 100°K at the 17-ID-2 FMX beamline (63) using a beam size of 1 × 1.5 µm and an EIGER 16M detector (DECTRIS) at the National Synchrotron Light Source II (NSLS II), Brookhaven National Laboratory. Diffraction data from APCDD1 crystal-form I were indexed, integrated, and scaled using the autoPROC toolbox (64), coupled with XDS (65), POINTLESS (66), and AIMLESS (67). Diffraction data from APCDD1 crystal-form II and eMBP-APCDD1 were indexed, integrated, and scaled using the XIA2 system (68), coupled with DIALS (69, 70) and POINTLESS (66). Diffraction anisotropy was further corrected using STARANISO (71). A randomly selected subset of 5% of the diffraction data was used as a cross-validation dataset to calculate *R*_free_.

### Structure Determination and Refinement.

The structure of eMBP-APCDD1 was determined by molecular replacement (MR) using PHASER (72) using the diffraction data scaled at the 30.0 to 2.5 Å resolution range without the anisotropy correction and using the MBP structure (PDB ID 3SET) as a template to obtain the initial phases. The resulting map contained three MBP copies that fit well into the electron density, whereas the electron density for APCDD1 was not interpretable. RoseTTAFold (28) was used to predict models of APCDD1, ABD1, and ABD2. All predicted models were superimposed to assess conserved core domains of ABD1 and ABD2 using the SSM algorithm of SUPERPOSE (73) in the CCP4 suite (74). Prior to MR, the values of Angstroms error estimates of the core domains were set to a constant value (=30 Å^2^) for the B-factors for all atoms. The second round of MR using PHASER (72) was conducted by fixing the position of the three MBP copies and using the core domains of ABD1 and ABD2 as templates to obtain the improved phases. The electron density was further improved after density modification with PARROT (75) and subsequently fed into BUCCANEER in the CCP4 suite (30, 74) for initial model building. The model of eMBP-APCDD1 was completed by manual building in COOT (76) and refinement was performed using REFMAC5 (77) and PHENIX Refine (78) with translation-libration-screw (TLS) parameterization. The resulting APCDD1 model from the eMBP-APCDD1 structure was used to determine the structures of APCDD1 crystal-form I and II by MR using PHASER (72). The subsequent model building and refinement were conducted using COOT (71) and PHENIX Refine (78) with TLS parameterization. Finally, the APCDD1 structures were built for crystal-form I (residues 50 to 473 for chain A and residues 50 to 468 for chain B), crystal-form II (residues 50 to 467 for chain A and residues 50 to 466 for chain B), and eMBP-APCDD1 (residues 47 to 468 for chain A, residues 47 to 469 for chain B, residues 47 to 472 for chain C, and residues 47 to 469 for chain D), except the regions where the electron densities were not interpretable for model building: crystal-from I (residues 389 to 391 and 406 to 408 for chain A; residues 193 to 195, 387 to 393, and 407 to 410 for chain B), crystal-form II (residues 175 to 178 and 391 to 394 for chain B), and eMBP-APCDD1 (residues 387 to 393 and 409 to 411 for chain A, 387 to 393 and 406 to 410 for chain C, and 389 to 390 and 409 to 414 for chain D). MOLPROBITY (79) was used to validate the models. The crystallographic statistics are listed in *SI Appendix*, Table S1.

### Structure Analysis.

Structure-based multiple sequence alignment was performed using Clustal Omega (53) and ESPript (80). Structure superposition was performed using the SSM algorithm of SUPERPOSE (73) in the CCP4 suite (74). The interior volume of the pocket was calculated using CASTp (29) with a 1.4 Å radius probe. Schematic 2D representations of protein and ligand interactions were generated using LigPlot+ (81). Searches for structure-based similarities to APCDD1, ABD1, and ABD2 were performed against the databases of the PDB and AlphaFold (36) using the DALI server (37). The computational model of APCDD1L based on APCDD1 was generated with Modeller (82). High-quality images of the molecular structures were generated with the PyMOL Molecular Graphic System (Version 2.5, Schrödinger, LLC). Schematic figures and other illustrations were prepared using GraphPad Prism (GraphPad Software, LLC) and Corel Draw (Corel Corporation). Structural biology applications used in this work were compiled and configured by SBGrid (83).

### Mass Spectrometry Analysis.

To identify the bound ligand in APCDD1 structures, HPLC coupled with mass spectrometry (MS) was used. Briefly, purified APCDD1 protein solution or conditioned media without APCDD1 (serving as a control for the compounds initially present) was mixed with the extraction solution (methyl tert-butyl ether/methanol/water in a ratio of 10:3:2.5) and the phases were separated. The organic phase was dried in a SpeedVac concentrator. The dried pellet was reconstituted in a solution of n-butanol and methanol in a 1:1 ratio. The sample was then analyzed by HPLC/MS on an Ultimate 3000 UPLC (using an Accucore C30 column) and Q-Exactive Plus Orbitrap. Ligand identification was performed by comparison of the mass spectrum of the analyte with the library database at Cayman Chemical.

### AP-Based Binding Assay.

For the AP fusion proteins (AP-APCDD1, APCDD1-AP, DKK1-AP, and AP-DKK1), HEK293T cells were grown in six-well plates and transfected with plasmids and PEI as described previously (27, 57). Conditioned media were collected 2 d posttransfection. For the biotinylated bait preparations, a 3:1:1 ratio of either LRP5-Avi or LRP6-Avi, MESD pHLsec (59), and pHLsec-BirA-ER (56) plasmids was transfected into HEK293T cells in the presence of 0.1 mM biotin (MilliporeSigma) and 4 mM valproic acid. Similarly, a 2.5:1.5:1 ratio of WNT7A, APCDD1-mV-Avi (or FZD4-mV-Avi, or empty vector, pLEXm), and pHLsec-BirA-ER (56) plasmids was transfected into HEK293T cells in the presence of 0.1 mM biotin and 4 mM valproic acid. The biotinylated baits were purified by the IMAC method (57) from 20 mL conditioned media collected 2 or 3 d posttransfection and eluted in 400 μL.

IMAC-concentrated complexes (100 μL each) were immobilized on 96-well streptavidin-coated plates (Thermo Fisher Scientific) at 4 °C overnight. For the detergent addition assays, 1% DDM (Anatrace), 0.1% DDM (Anatrace), or 2.25% Triton X-114 (MilliporeSigma) were included in all steps from the 96-well plate immobilization to the washes. The wells were then washed three times with wash buffer [10 mM HEPES, pH 7.5, 0.15 M NaCl, 0.05% (w/v) Tween-20] supplemented or not with the indicated detergents and incubated with a 10-fold dilution of bovine serum albumin (BSA) blocker buffer (Thermo Fisher Scientific 37525) in wash buffer for 1 h at 25 °C. The wells were washed with wash buffer with the indicated detergents and incubated with conditioned media containing AP probes (APCDD1-AP, AP-APCDD1, DKK-AP, or AP-DKK) or recombinant Nb-AP proteins at 4 °C overnight. The wells were subsequently washed three times with wash buffer with the indicated detergents and incubated with BluePhos phosphatase substrate solution (Kirkegaard and Perry Laboratories 50-88-00) to visualize the bound AP probes. The binding assays were performed twice.

Biotinylated baits were immobilized on 96-well streptavidin-coated plates (Thermo Fisher Scientific) at 4 °C overnight. The wells were then washed three times with wash buffer [10 mM HEPES, pH 7.5, 0.15 M NaCl, 0.05% (w/v) Tween-20] and incubated with a 10-fold dilution of BSA blocker buffer (Thermo Fisher Scientific 37525) in wash buffer for 1 h at 25 °C. The wells were washed with wash buffer and incubated with conditioned media containing AP probes (APCDD1-AP, AP-APCDD1, DKK-AP, or AP-DKK) or recombinant Nb-AP proteins at 4 °C overnight. The wells were subsequently washed three times with wash buffer and incubated with BluePhos phosphatase substrate solution (Kirkegaard and Perry Laboratories 50-88-00) to visualize the bound AP probes.

## Supplementary Material

Appendix 01 (PDF)

## Data Availability

X-ray structure data have been deposited in Protein Data Bank (8E0P, 8E0R, and 8E0W) for eMBP-APCDD1, APCDD1 crystal-form I and form II, respectively (84–86).
