# Supplementary material for: Structure of WNT inhibitor adenomatosis polyposis coli down-regulated 1 (APCDD1), a cell-surface lipid-binding protein
Source: Proc Natl Acad Sci U S A. 2023 May 8;120(20):e2217096120. doi: 10.1073/pnas.2217096120 (PMC10193966; doi:10.1073/pnas.2217096120)
Supplement: Supplementary file 1 — Appendix 01 (PDF) [file pnas.2217096120.sapp.pdf]

Supplementary information for

Structure of Wnt inhibitor APCDD1, a Cell-Surface Lipid-Binding Protein

Fu-Lien Hsieh[1,2]†, Tao-Hsin Chang[1,2]†*, Sandra B. Gabelli[3,4,5]‡, Jeremy Nathans[1,2,6,7]*

[1]Department of Molecular Biology and Genetics
[2]Howard Hughes Medical Institute
[3]Department of Biophysics and Biophysical Chemistry
[4]Department of Medicine
[5]Department of Oncology
[6]Department of Neuroscience
[7]Wilmer Eye Institute; Johns Hopkins University School of Medicine, Baltimore, 21205, United States.

†These authors contributed equally to this work

*Corresponding authors. Email: taohsin.chang@gmail.com; jnathans@jhmi.edu

‡Present address: Merck Research Laboratories, 770 Sumneytown Pike, West Point, PA 19486

References

1. Liebschner D*, et al.* (2019) Macromolecular structure determination using X-rays, neutrons and electrons: recent developments in Phenix. *Acta Crystallogr D Struct Biol* 75(Pt 10):861-877.
2. Tian W, Chen C, Lei X, Zhao J, & Liang J (2018) CASTp 3.0: computed atlas of surface topography of proteins. *Nucleic Acids Res* 46(W1):W363-W367.
3. Sievers F & Higgins DG (2014) Clustal omega. *Curr Protoc Bioinformatics* 48:3 13 11-16.
4. Robert X & Gouet P (2014) Deciphering key features in protein structures with the new ENDscript server. *Nucleic Acids Res* 42(Web Server issue):W320-324.
5. Kumar S, Stecher G, & Tamura K (2016) MEGA7: Molecular Evolutionary Genetics Analysis Version 7.0 for Bigger Datasets. *Mol Biol Evol* 33(7):1870-1874.
6. Saitou N & Nei M (1987) The neighbor-joining method: a new method for reconstructing phylogenetic trees. *Mol Biol Evol* 4(4):406-425.
7. Eswar N*, et al.* (2006) Comparative protein structure modeling using Modeller. *Curr Protoc Bioinformatics* Chapter 5:Unit-5 6.
8. Sabbagh MF*, et al.* (2018) Transcriptional and epigenomic landscapes of CNS and non-CNS vascular endothelial cells. *Elife* 7.

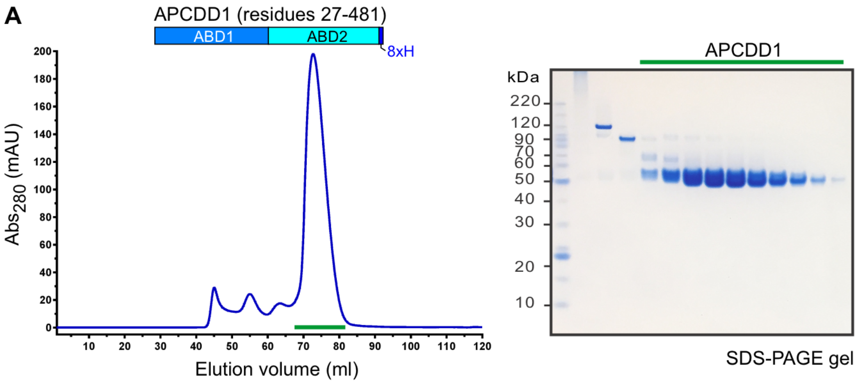

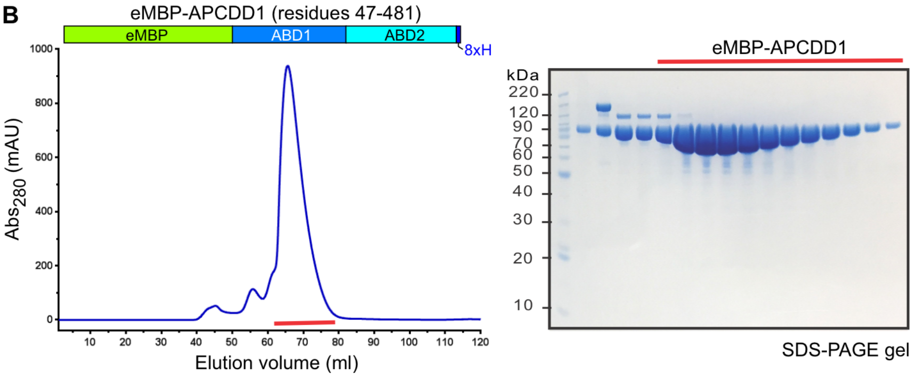

Fig. S1. Protein purification of APCDD1 and eMBP-APCDD1.
(**A**) Size exclusion chromatography (SEC) elution profile and SDS-PAGE electrophoresis of the extracellular domain (ECD) of APCDD1. The peak fractions are marked with a green line. (**B**) SEC elution profile and SDS-PAGE electrophoresis of eMBP-APCDD1, with peak fractions marked with a red line.

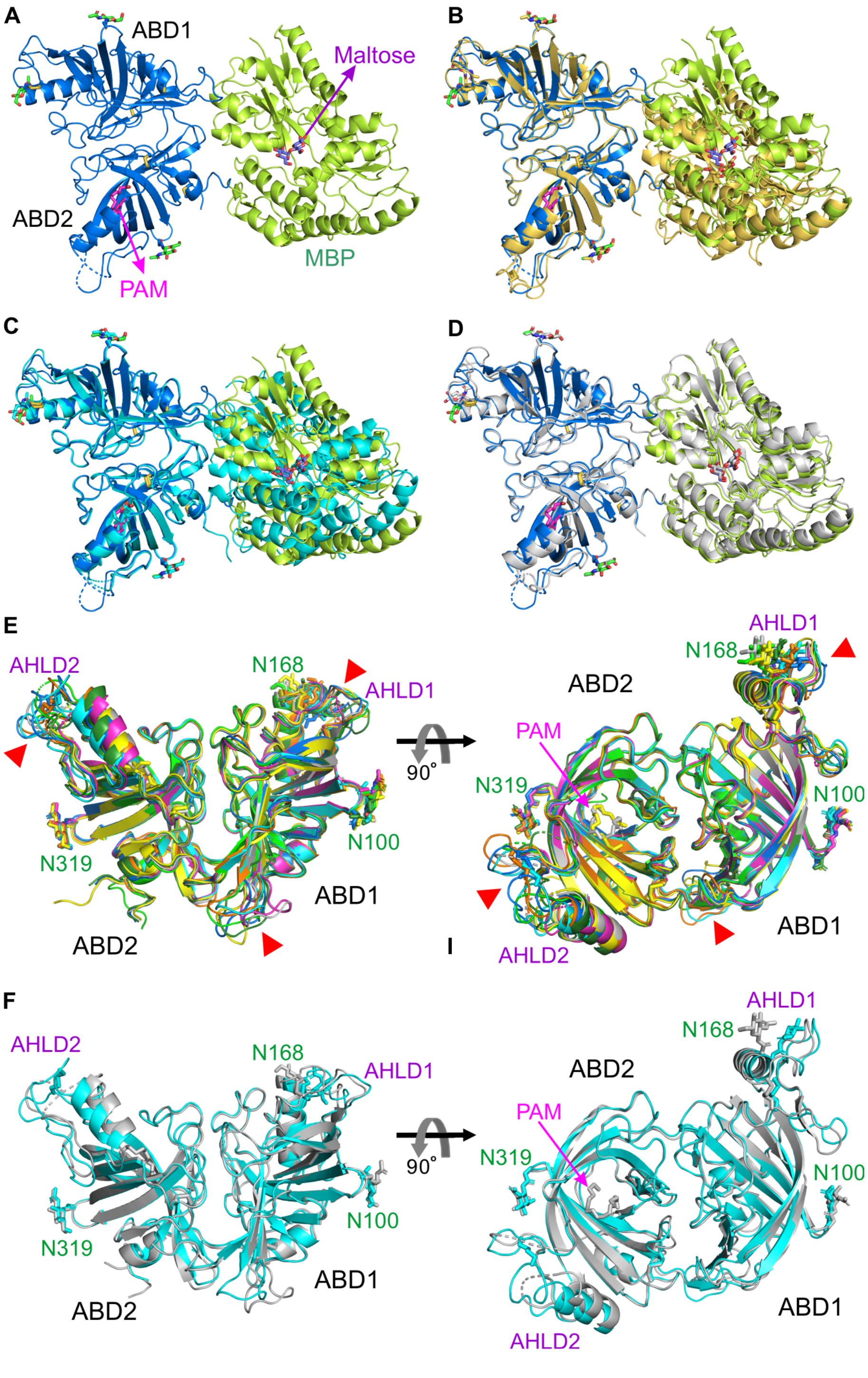

Fig. S2. Comparison among four eMBP-APCDD1 structures and structural analyses displaying immobile and flexible regions of APCDD1.
(**A**) The structure of eMBP-APCDD1 (chain A), with APCDD1 in blue and eMBP in green. PAM (magenta) and maltose (purple) are shown as sticks. (**B**) Superposition of eMBP-APCDD1 chains A (blue) and B (gold) based on the alignment of the APCDD1 portion of the fusion protein shows the domain mobility between APCDD1 and MBP. The r.m.s. deviation between the two APCDD1 structures is 0.68 Å over 405 Cα atoms. (**C**) Superposition of eMBP-APCDD1 chains A (blue) and C

(cyan) as in (B) shows domain mobility between APCDD1 and MBP with a r.m.s. deviation of 0.63 Å over 403 APCDD1 Cα atoms. (**D**) Superposition of eMBP-APCDD1 chains A (blue) and D (gray) as in (B) reveals a similar domain arrangement with a r.m.s. deviation of 0.75 Å over 498 APCDD1 Cα atoms. (**E**) Ribbon diagram showing a superposition of APCDD1 molecules from APCDD1 and eMBP-APCDD1 crystal structures. Green, crystal-form I chain A; magenta, crystal-form I chain B; cyan, crystal-form II chain A; orange, crystal-form II chain B; gray, eMBP-APCDD1 chain A; blue, eMBP-APCDD1 chain B; yellow, eMBP-APCDD1 chain C; forest, eMBP-APCDD1 chain D. Flexible regions are highlighted with red triangles. Three regions – AHLD1, AHLD2, and the connecting loop between ABD1 and ABD2 – show structural flexibility. The immobile regions include the structure and relative orientation of the β-barrel domains. N-linked glycans and PAM are shown as sticks. See Table S2 for statistics on the pairwise superpositions. (**F**) Superposition of the two most conformationally distinct APCDD1 structures – APCDD1 in apo-form (crystal-form II chain A; cyan) and eMBP-APCDD1 with bound PAM (chain C; gray) – reveals a r.m.s. deviation of 1.14 Å over 400 Cα atoms.

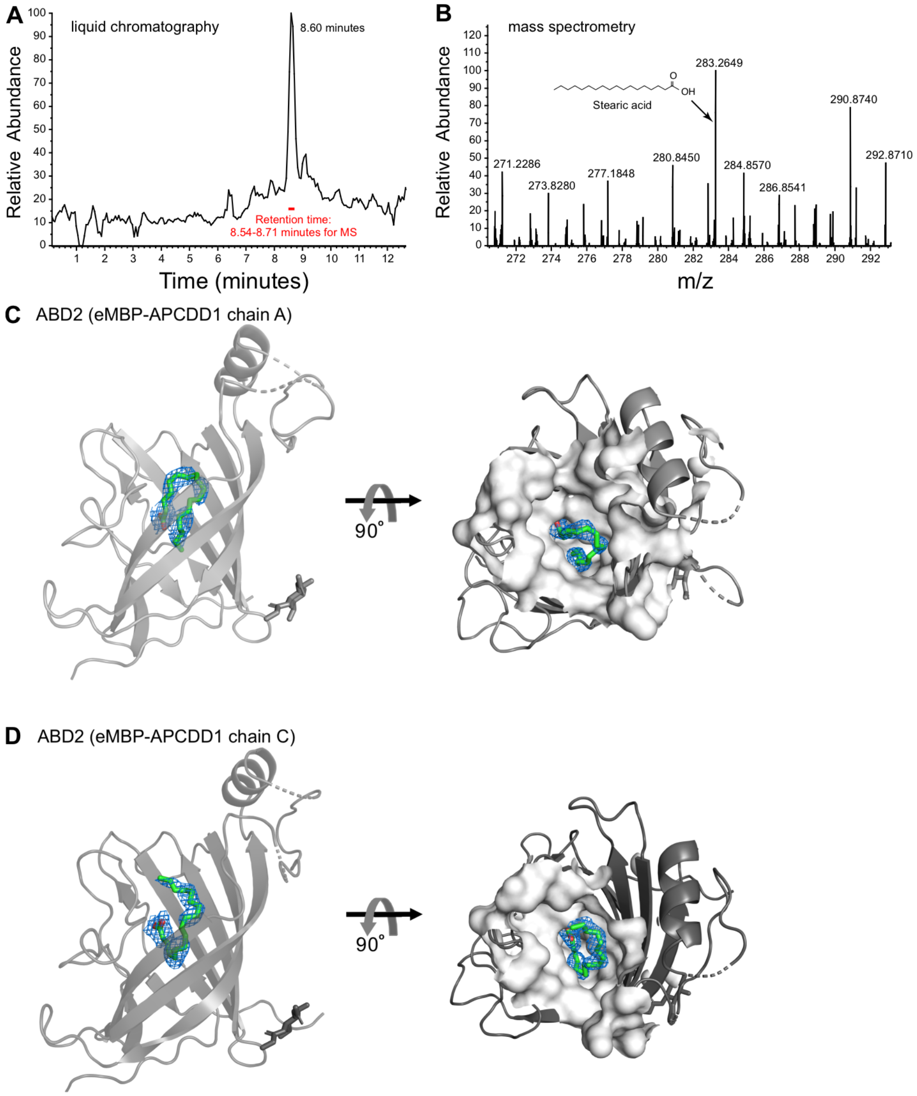

Fig. S3. Lipid identification by mass spectrometry and the interaction between ABD2 and stearic acid.

(**A**) Lipids were extracted from the APCDD1 preparation used for crystallization (fig. S1A) and analyzed by high-performance liquid chromatography (HPLC). (**B**) The HPLC peak indicated in panel (**A**) was identified as stearic acid (an 18-carbon lipid) by mass spectrometry. (**C**) The structure of ABD2 (eMBP-APCDD1 chain A; gray ribbon representation) in complex with stearic acid (green sticks) after refinement in PHENIX (1). The 2|F_O|-|F_C| electron density (blue meshes) of stearic acid is contoured at the 0.9 σ level. The hydrophobic pocket of ABD2 is shown as a gray surface representation. (**D**) The structure of ABD2 (eMBP-APCDD1 chain C; gray ribbon representation) in complex with stearic acid (green sticks) after refinement in PHENIX (1), as described for panel (**C**). The orientation and conformation of the stearic acid models are almost identical to the PAM models, as shown in Fig. 4B and C.

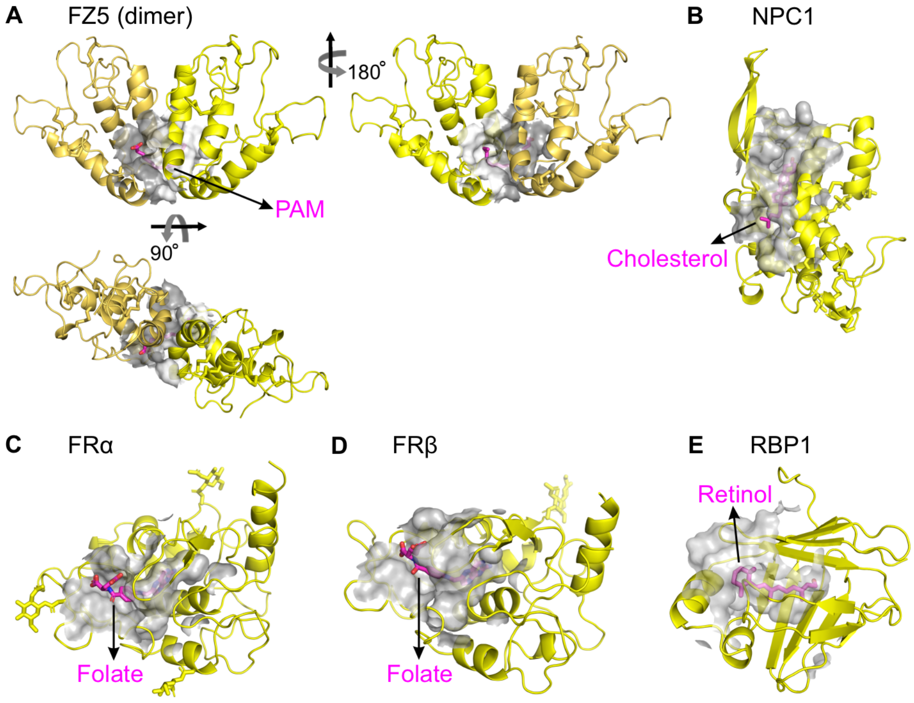

Fig. S4. Structural analyses of hydrophobic ligand-binding pockets of FZ5, NPC1, FRα, FRβ, and RBP1.

Surface representations (gray) and hydrophobic ligands (magenta sticks), rendered with CASTp (2), are shown for the following lipid-binding pockets. (**A**) FZ5 dimer (gold and yellow) in complex with PAM (PDB ID 5URY). (**B**) Niemann-Pick C1 protein (NPC1) in complex with cholesterol (PDB ID 3GKI). (**C**) Folate receptor α (FRα) in complex with folate (PDB ID 4LRH). (**D**) Folate receptor β (FRβ) in complex with folate (PDB ID 4KMZ). (**E**) Retinol-binding protein (RBP1) in complex with retinol (PDB ID 5HBS).

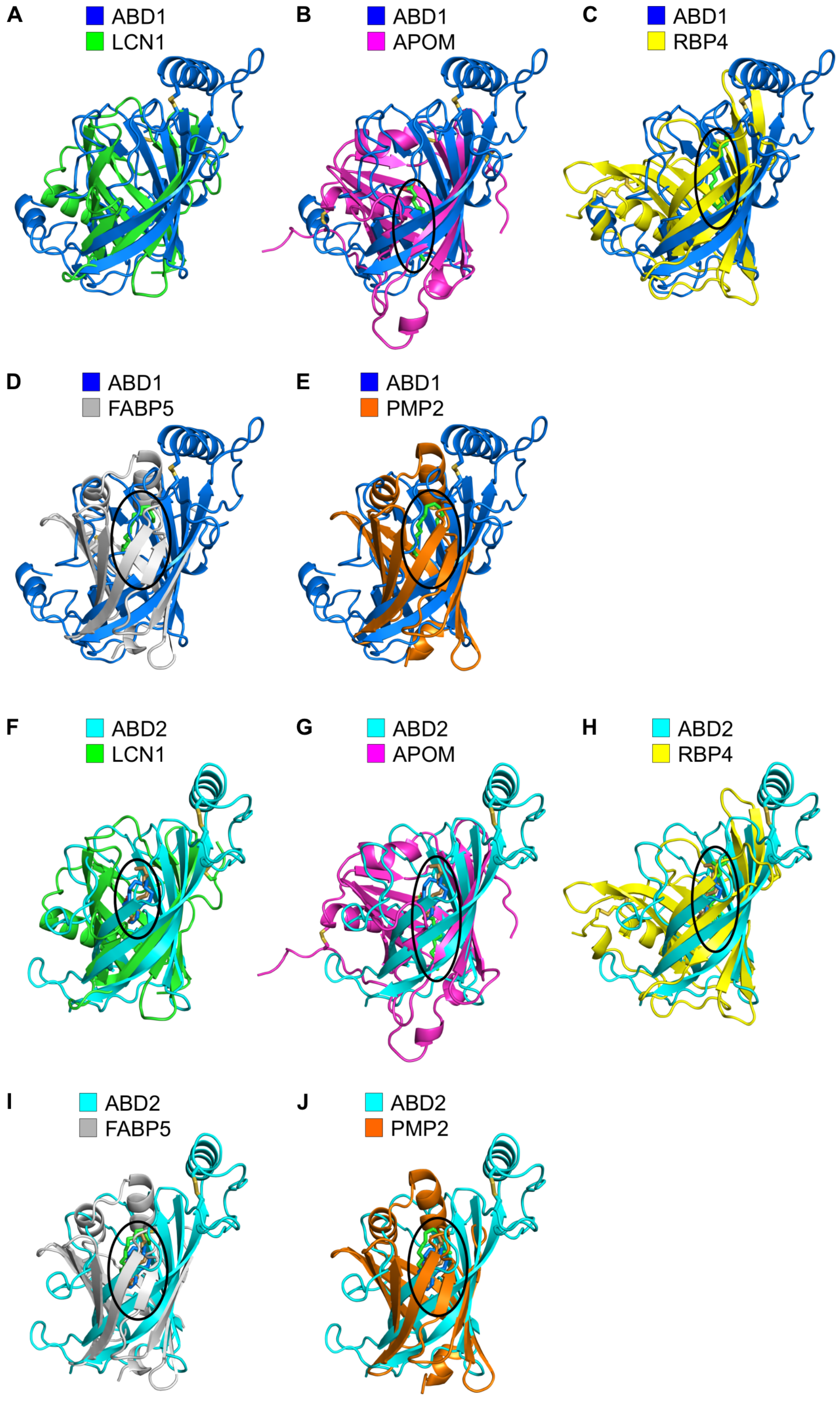

Fig. S5. Structural comparison of APCDD1 and the lipocalin and P2 families of hydrophobic ligand-binding proteins.
(**A**) Superposition of ABD1 (blue) and human lipocalin-1 (LCN1; green; PDB ID 3EYC) shows a r.m.s. deviation of 3.65 Å over 120 Cα atoms. (**B**) Superposition of ABD1 (blue) and human apolipoprotein

M (APOM; magenta) with bound myristic acid (green stick, highlighted by a circle) (PDB ID 2WEW) shows a r.m.s. deviation of 3.31 Å over 101 Cα atoms. (**C**) Superposition of ABD1 (blue) and human retinol-binding protein 4 (RBP4; yellow) with bound retinol (green stick, highlighted by a circle) (PDB ID 1BRP) reveals a r.m.s. deviation of 3.31 Å over 104 Cα atoms. (**D**) Superposition of ABD1 (blue) and human fatty acid-binding protein 5 (FABP5; gray) with bound palmitate (green stick, highlighted by a circle) (PDB ID 1B56) shows a r.m.s. deviation of 2.86 Å over 81 Cα atoms. (**E**) Superposition of ABD1 (blue) and human peripheral myelin protein 2 (PMP2; orange) with bound palmitate (green stick, highlighted by a circle) (PDB ID 2WUT) shows a r.m.s. deviation of 2.56 Å over 76 Cα atoms. (**F-J**) ABD2 (cyan) with bound PAM (blue and gold sticks for two PAM conformations, respectively) was superposed on the following structures. **(F)** LCN1 (green) shows a r.m.s. deviation of 3.46 Å over 107 Cα atoms, (**G**) APOM (magenta) with bound myristic acid (green stick) shows a r.m.s. deviation of 3.52 Å over 98 Cα atoms, (**H**) RBP4 (yellow) with bound retinol (green stick) shows a r.m.s. deviation of 3.07 Å over 97 Cα atoms, (**I**) FABP5 (gray) with bound palmitate (green stick) shows a r.m.s. deviation of 2.48 Å over 78 Cα atoms, and (**J**) PMP2 (orange) with bound palmitate (green stick) shows a r.m.s. deviation of 2.43 Å over 77 Cα atoms. The binding site for ligands is highlighted by a circle.

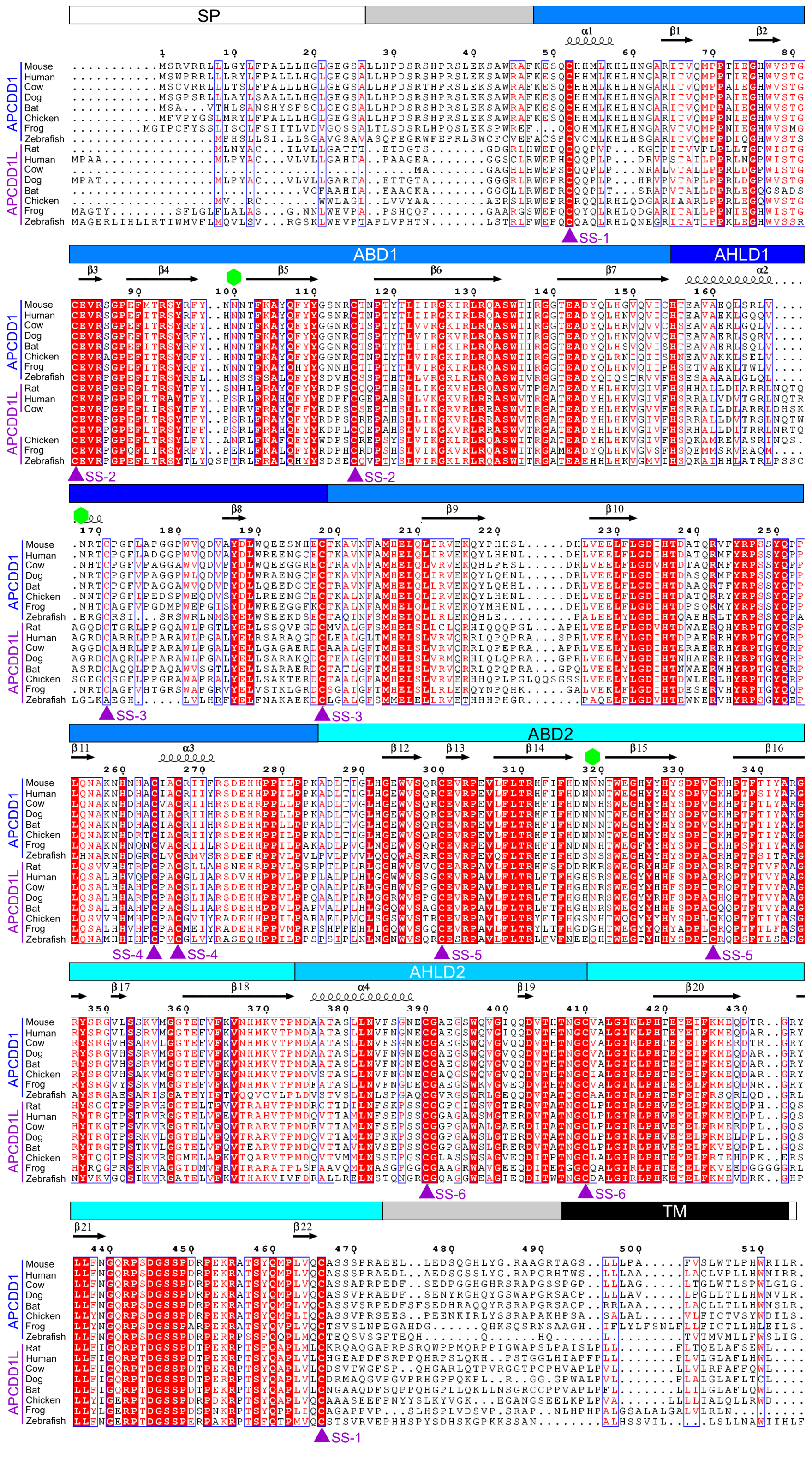

S6. Multiple sequence alignment of APCDD1 and APCDD1L.

Protein sequences of APCDD1 from mouse (NCBI accession: NP_573500.2), human (NCBI accession: NP_694545.1), cow (UniPort code: F1MNJ4), dog (NCBI accession: XP_537333), bat (UniPort code:

G1NWM1), chicken (UniPort code: Q5R2I8), frog (UniPort code: Q66KI8), and zebrafish (UniPort code: A0A0G2L4I0) and APCDD1L from rat (NCBI accession: XP_006235773), human (NCBI accession: NP_699191), cow (NCBI accession: XP_024856930), dog (NCBI accession: XP_038290136), bat (UniPort code: G1P719), chicken (NCBI accession: XP_015152029), frog (NCBI accession: XP_031750509), and zebrafish (UniPort code: E7F3Y0) are aligned using Clustal Omega (3) and formatted using ESPript (4). Secondary structure elements and numbering for mouse APCDD1 are shown above the sequences. The positions of cysteines and their disulfide bonds (SS-1 to SS-6) are indicated by purple triangles. The signal peptide (SP), presumptive non-structural regions (gray), ABD1, AHLD1, ABD2, AHLD2, and the transmembrane domain (TM) are marked above the aligned sequences. N-linked glycosylation sites are denoted by green hexagons.

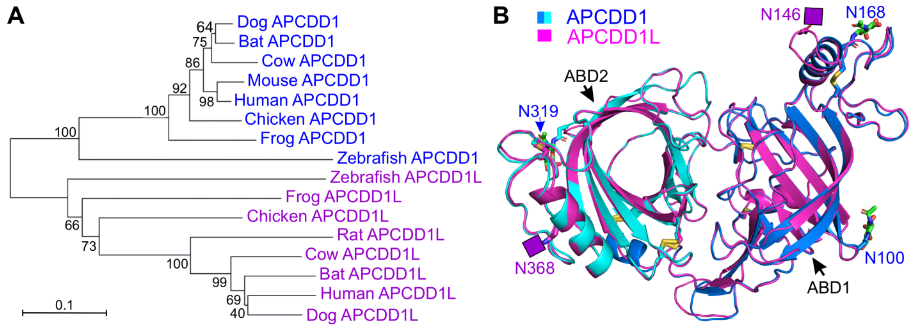

Fig. S7. Phylogenetic analysis and structural comparison of APCDD1 with the predicted structure of APCDD1L.

(**A**) Phylogenetic analysis of APCDD1 and APCDD1L was conducted with MEGA7 (8) using the neighbor-joining method (6). The tree was drawn to scale, with branch lengths in the same units as those of the evolutionary distances used to infer the phylogenetic tree. (**B**) Mouse APCDD1 and rat APCDD1L have 46.1% amino acid sequence identity (see also Fig. S6). Comparison between APCDD1 (blue for ABD1 and cyan for ABD2) structure and the predicted mouse APCDD1L (magenta) structure, generated by homology modeling with MODELLER (7), showed a r.m.s. deviation of 0.17 Å over 339 Cα atoms. N-linked glycans of APCDD1 are shown as sticks and putative N-linked glycans of APCDD1L are marked with purple squares.

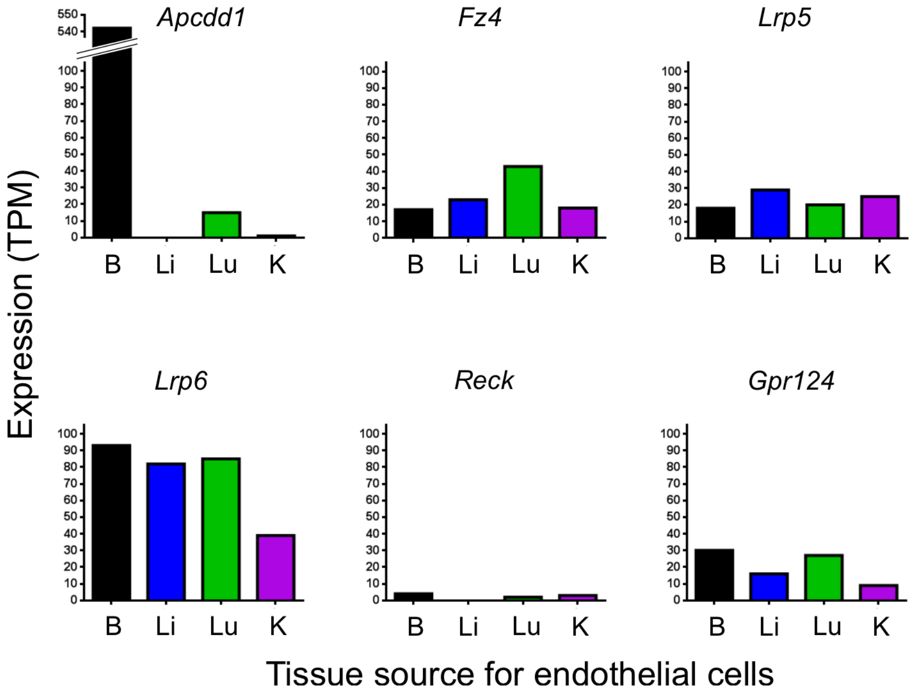

Fig. S8. RNA-seq reveals endothelial cell transcripts for major WNT signaling components. Expression levels (transcripts-per-million, TPM) based on RNA-seq of FACS-purified mouse endothelial cells from brain (B), liver (Li), lung (Lu), and kidney (K), all harvested at postnatal day 7, for the indicated genes (Apcdd1, Fz4, Lrp5, Lrp6, Reck, and GPR124) (8). RNA-seq expression plots were rendered with VECTRDB (8).
